# Biocontrol potential of wine yeasts against four grape phytopathogenic fungi disclosed by time-course monitoring of inhibitory activities

**DOI:** 10.3389/fmicb.2023.1146065

**Published:** 2023-03-07

**Authors:** Marcos Esteves, Patrícia Lage, João Sousa, Filipe Centeno, Maria de Fátima Teixeira, Rogério Tenreiro, Ana Mendes-Ferreira

**Affiliations:** ^1^WM&B—Laboratory of Wine Microbiology and Biotechnology, Department of Biology and Environment, University of Trás-os-Montes and Alto Douro, Vila Real, Portugal; ^2^BioISI—Biosystems & Integrative Sciences Institute, Faculty of Sciences, University of Lisbon, Lisbon, Portugal; ^3^PROENOL—Indústria Biotecnológica, Lda, Canelas, Portugal

**Keywords:** biocontrol, wine yeasts, phytopathogenic fungi, antagonism, *Aspergillus niger*, *Botrytis cinerea*, *Penicillium* sp., *Rhizopus* sp.

## Abstract

Grapes’ infection by phytopathogenic fungi may often lead to rot and impair the quality and safety of the final product. Due to the concerns associated with the extensive use of chemicals to control these fungi, including their toxicity for environment and human health, bio-based products are being highly preferred, as eco-friendlier and safer alternatives. Specifically, yeasts have shown to possess antagonistic activity against fungi, being promising for the formulation of new biocontrol products.In this work 397 wine yeasts, isolated from Portuguese wine regions, were studied for their biocontrol potential against common grapes phytopathogenic fungal genera: *Aspergillus*, *Botrytis*, *Rhizopus* and *Penicillium*. This set comprised strains affiliated to 32 species distributed among 20 genera. Time-course monitoring of mold growth was performed to assess the inhibitory activity resulting from either diffusible or volatile compounds produced by each yeast strain. All yeasts displayed antagonistic activity against at least one of the mold targets. *Rhizopus* was the most affected being strongly inhibited by 68% of the tested strains, followed by *Botrytis* (20%), *Aspergillus* (19%) and *Penicillium* (7%). More notably, the approach used allowed the detection of a wide array of yeast-induced mold response profiles encompassing, besides the decrease of mold growth, the inhibition or delay of spore germination and the complete arrest of mycelial extension, and even its stimulation at different phases. Each factor considered (taxonomic affiliation, mode of action and fungal target) as well as their interactions significantly affected the antagonistic activity of the yeast isolates. The highest inhibitions were mediated by volatile compounds. Total inhibition of *Penicillium* was achieved by a strain of *Metschnikowia pulcherrima*, while the best performing yeasts against *Rhizopus*, *Aspergillus* and *Botrytis*, belong to *Lachancea thermotolerans*, *Hanseniaspora uvarum* and *Starmerella bacillaris*, respectively. Notwithstanding the wide diversity of yeasts tested, only three strains were found to possess a broad spectrum of antagonistic activity, displaying strong or very strong inhibition against the four fungal targets tested. Our results confirm the potential of wine yeasts as biocontrol agents, while highlighting the need for the establishment of fit-for-purpose selection programs depending on the mold target, the timing, and the mode of application.

## 1. Introduction

Phytopathogenic fungi are one of the major concerns in several agricultural crops, being responsible for significant losses in production and associated economy. Grapes are a particularly rich nutrient source for pathogenic agents, whose action can affect not only grape health and productivity, but also the quality of the final product, whether they are intended for fresh human consumption or for processed products such as raisins, juices and wines. Necrotrophic filamentous fungi are the group of microorganisms presenting most of the concerns in the pre- and post-harvested grapes. If not timely controlled, several species mainly belonging to the genera *Alternaria*, *Aspergillus*, *Botrytis*, *Fusarium*, *Mucor*, *Penicillium* and *Rhizopus* are capable of causing major grape diseases involving the decay of fruit, production of mycotoxins and/or off-flavors (Kassemeyer, 2017; Solairaj et al., 2021). In wine industry, grapes infected with molds are more prone to rot and harbor much higher loads of microorganisms, particularly yeasts and bacteria, that may lead to microbial wine spoilage (Barata et al., 2012). To avoid fungal infections, table grape-growers and winemakers mostly rely on the application of synthetic fungicides on-field, during the different phenological growth stages of the grapes, and at postharvest. However, the increasing concerns about on the harmful side-effects of these compounds on environment and human health, along with the discover of multi-drug resistant biotypes of fungal pathogens, has encouraged scientific community to find efficient, less toxic and more sustainable resources (Wilson and Wisniewski, 1989; Pertot et al., 2017a). In this context, the concept of biocontrol emerged and currently is restricted to the use of living agents (including viruses), with the ability of preventing or at least reduce the growth of the pathogen or the disease producing activity (Ward, 2016; Stenberg et al., 2021).

Among these biocontrol agents, several yeasts have demonstrated antagonistic activity against different fungal phytopathogens of fruits and vegetables (reviewed in Ferraz et al., 2019; Freimoser et al., 2019; Di Canito et al., 2021). Competition for nutrients and space, antibiosis (production of diffusible or volatile compounds), mycoparasitism, and induction of host resistance have been pointed out as the main modes of action underlying their antagonistic activity (reviewed in Bélanger et al., 2012; Spadaro and Droby, 2016). Therefore, it is likely that the best sources of antagonistic strains are their own natural environments in which they have developed strategies to colonize, access nutrients and space and, to inhibit other coexisting microorganisms (including epiphytic pathogens), thus ensuring their survival (Parafati et al., 2015; Pretscher et al., 2018; Pereyra et al., 2021).

In this line, the wine ecosystem (grapes and leaves surfaces, grape-juices, wines and winery equipment) constitutes a valuable source of strains with potential to be used in the biocontrol of grape phytopathogens as it harbors a large diversity of yeast species. The most frequently reported yeasts associated with wine-related ecosystems either belong to oligotrophic and oxidative species affiliated to genera such as *Aureobasidium*, *Cryptococcus*, *Filobasidium*, *Naganishia*, *Rhodotorula* and *Sporidiobolus* as well as to copiotrophic and/or fermentative species of genera such as *Candida*, *Hanseniaspora*, *Metschnikowia*, *Meyerozyma*, *Pichia*, *Saccharomyces*, *Starmerella*, *Torulaspora* and *Wickerhamomyces* (Barata et al., 2012; Bokulich et al., 2014; Sirén et al., 2019).

The prospect of using these so-called “wine yeasts” as antagonists of grape fungal pathogens led to a variety of *in vitro* screenings using dual-culture assays (Suzzi et al., 1995; Bleve et al., 2006; Raspor et al., 2010; Nally et al., 2012; Pantelides et al., 2015; Lemos Junior et al., 2016; Cordero-Bueso et al., 2017; Pretscher et al., 2018; Reyes-bravo et al., 2019). In addition to the assessment of their inhibitory activity, and according to the experimental setup used, this screening methodology also gave insights on the potential modes of action associated, typically the production of antimicrobial compounds (Köhl et al., 2019). In one of the first reports (Suzzi et al., 1995), natural wine yeast strains of the genera *Saccharomyces* and *Zygosaccharomyces* were found to be antagonists of 10 species of soil-borne fungal plant pathogens. Later, *Aspergillus carbonarius* and *A. niger* were found to be inhibited by strains of *Pic. kudriavzevii*, *Met. pulcherrima*, *L. thermotolerans*, *Pic. terricola* and *Can. incommunis* (Bleve et al., 2006). Studies targeting the biocontrol of *Botrytis cinerea* have identified strains of *Aur. pullulans*, *Met. pulcherrima* and *Mey. guilliermondii* (Raspor et al., 2010); of *Schizosaccharomyces pombe* and *Sac. cerevisiae* (Nally et al., 2012); and *Sta. bacillaris* (Lemos Junior et al., 2016) with antagonistic activity. Cordero-Bueso et al. (2017) also identified strains of *H. uvarum*, *Mey. guilliermondii*, *Pic. kluyveri*, *Sac. cerevisiae*, *Han. clermontiae*, *Met. pulcherrima*, and *Can. californica* with a strong antagonistic action against *B. cinerea*, *A. carbonarius* and *P. expansum*. More recently, Reyes-bravo et al. (2019) reported strains of *Aur. pullulans, Cry. antarcticus*, *Cry. terrestris* and *Cry. oeirensis* capable of inhibit the growth of both *B. cinerea* and *P. expansum*. The biocontrol activity of several of the wine yeast strains above enumerated have also been validated *in vivo*, using wounded grapes (Bleve et al., 2006; Raspor et al., 2010; Nally et al., 2012; Lemos Junior et al., 2016; Cordero-Bueso et al., 2017).

In this context, the present work aimed to evaluate the antagonistic activity of a wide-range of yeast strains, previously isolated from grapes, grape-juices and wines, against fungi belonging to *Aspergillus*, *Botrytis*, *Rhizopus* and *Penicillium* genera. A time-course assessment of the yeasts’ effects on the growth of the targeted fungi, associated with the production of diffusible and volatile compounds, was conducted in order to get insights into the yeast-mold target interaction and the mode of action, to ultimately uncover more suitable biocontrol agents for application in the vineyards and/or at postharvest.

## 2. Materials and methods

### 2.1. Microorganisms and culture conditions

Three hundred and ninety-seven yeast isolates, randomly selected from a wider yeast culture collection available at WMB LAB at UTAD assembled over the last 20 years and comprising more than 3,000 microbial isolates collected from wine-related environments from different wine-producing regions across Portugal, were used. Yeast isolates were stored in stocks at −80°C in YPD medium (5 g/L of yeast extract, 10 g/L of peptone, 20 g/L of glucose) added with 20% (v/v) glycerol. The selected isolates were transferred to YPD agar (5 g/L of yeast extract, 10 g/L of peptone, 20 g/L of glucose, 20 g/L of agar), grown at 28°C and maintained at 4°C.

Four strains of phytopathogenic fungal genera were used, namely *Aspergillus niger* strain AN1 (isolated from wine bottle cork), *Botrytis cinerea* strain BO1 (isolated from table grapes)*, Rhizopus* sp. strain MU3 (isolated from wine grapes) and *Penicillium* sp. strain PE3 (isolated from wine grapes). Stocks of mold cultures were prepared in Potato Dextrose Agar (PDA) slants flooded in liquid paraffin and maintained at room temperature. Prior to their use, mold cultures were transferred to PDA plates and grown at 25°C. Spore suspensions of each fungal strain were obtained by harvesting spore mass at the surface of 15 days old colonies with glass beads using PBS buffer 1X with 0.01% Tween 20 and 50% (v/v) glycerol, to assist in spore dispersal and ultra-freezing preservation. After filtration through sterile glass wool, spore concentration was determined using a hemocytometer, adjusted to 1 × 10^6^ or 1 × 10^4^ spores/mL for the ascomycetous phytopathogens and *Rhizopus* sp., respectively. Spore suspensions were divided into aliquots and stored at −80°C.

### 2.2. Yeast molecular identification and characterization

Genomic DNA extraction of the autochthonous isolates was performed by phenol-chloroform-isoamyl alcohol method from single-strain pure cultures (Seixas et al., 2019). The identification was achieved by 26S rDNA D1/D2 sequencing using primers NL-1 (5′-GCA TAT CAA TAA GCG GAG GAA AAG-3′) and NL-4 (5′-GGT CCG TGT TTC AAG ACG G-3′), and subsequent BLAST comparison with the deposited 26S rRNA gene sequences in the GenBank data library. Species identification was attributed to an isolate if its 26S rDNA D1/D2 sequence diverged by no more than 2–3 base substitutions (i.e., ≥99.0% sequence identity) to that of a taxonomically accepted species type strain. All isolates matching to *Metschnikowia pulcherrima-*clade species were affiliated to *Met. pulcherrima* as proposed by Sipiczki, (2022). Differentiation of *Han. opuntiae* and *Han. guilliermondii* was achieved by 5.8S-ITS-RFLP analysis, in which ITS1/ITS4 amplification products were digested with restriction enzyme DraI, and separated on 2% agarose gel on 1 × TBE buffer as previously described by Nisiotou and Nychas, (2007).

The molecular diversity of yeast isolates belonging to the most prevalent genera was also evaluated through PCR-fingerprinting, using minisatellite csM13 (5′GAGGGTGGCGGTTCT3′) and microsatellite (GTG)_5_ (5′GTGGTGGTGGTGGTG3′), as single primers, following the methodology described previously by Barbosa et al. (2018). DNA banding patterns were analyzed using the BioNumerics software (version 5.0, Applied Maths, Sint-Martens-Laterat, Belgium). Similarities among isolates, using the combined (GTG)_5_ and csM13 genomic fingerprinting patterns, were estimated using the Pearson coefficient and clustering was based on the UPGMA method. A cut-off value of 80% similarity was used to define the number of clusters inside each genus.

### 2.3. Extracellular enzymatic activities of yeasts

The ability of the set of yeast strains to secrete lytic enzymes (protease, pectinase, chitinase, glucanase, cellulase, mannanase, and amylase) eventually associated with the suppression of growth of fungal pathogens was evaluated by spotting 3 μL of freshly grown yeast culture suspensions (~10^7^ cells/mL) onto solid media containing the specific enzyme substrates.

For proteolytic activity, Skim Milk agar was used. The formation of a light halo around the colonies after incubation at 28°C for 5 days indicated the enzymatic activity (Strauss et al., 2001).

Pectinolytic activity was tested by using a selective medium containing 12.5 g/L polygalacturonic acid, 6.8 g/L potassium phosphate (pH 3.5), 6.7 g/L yeast nitrogen base without ammonium sulfate, 10 g/L glucose, and 20 g/L agar. After incubation at 28°C for 5 days, the plates were stained with 0.1% (w/v) ruthenium red after the colonies being rinsed off with distilled water. Colonies showing a purple halo were considered positive (Strauss et al., 2001).

For glucanase, chitinase and cellulase activities YPD plates containing 0.2% β-D-glucan from barley or 0.2% chitin from shrimp shells or carboxymethyl cellulose (CMC) sodium salt were used, respectively. After incubation, at 28°C for 5 days, the plates were rinsed with distilled water and stained with 0.03% (w/v) Congo red. A clear zone around the colony was indicative of the presence of glucanase or chitinase activity (Strauss et al., 2001). For the cellulase assay, after incubation, the plates for were stained with 0.1% (w/v) Congo red solution for 30 min and then washed with 1 M NaCl solution for 15 min. The detection of a degradation halo around the colonies was indicative of a positive result (Chen et al., 2018).

The endo-1,4-β-mannanase activity was determined using a medium containing 9.0 g/L peptone, 1.0 g/L yeast extract, 1.0 g/L KH_2_PO_4_, 0.5 g/L MgSO_4_.7H_2_O, 15 g/L agar and a carbon source of commercial mannan, azo-carob-galactomannan (2 g/L). The pH of the medium was adjusted to 5.5. Positive mannanase producers were detected based on the clear zone formed on the medium around the colony after 5 days of incubation at 28°C (Asfamawi et al., 2013).

Amylase activity was assessed using a selective medium containing 10 g/L of yeast extract, 20 g/L of peptone from meat, 20 g/L of starch, and 20 g/L of agar. The pH of the medium was adjusted to 5.0. The amylase plates were overlaid with Lugol’s solution (iodine–potassium iodide solution) and amylase activity was detected by the presence of a bright lysis zone around the colonies, while the rest of the medium remained violet, as the dye solution stained the non-hydrolyzed starch (Pretscher et al., 2018).

### 2.4. *In vitro* antagonistic activity assays

The antagonistic activity of the set of 397 yeast strains against four common phytopathogenic fungal agents was evaluated using two *in vitro* assays approaches related to distinct modes of action underlying their inhibition. For all the assays, yeast strains were pre-cultivated in YPD medium and incubated overnight at 28°C. Prior to inoculation of each mold target, the suspensions of spores, prepared as described above, were removed from the freezer, and allowed to attain room temperature. The incubation period and the measuring points were previously established by performing mock trials in which the time taken for each mold target to extend from the point of inoculation to the edge of the Petri dish was observed. Based on these experiments it was established, a total incubation period of 40 h and 4, 5, and 7 days for *Rhizopus* sp., *B. cinerea*, *A. niger* and *Penicillium* sp., respectively, and daily measurements of radial mycelial extension. Due to the rapid growth of *Rhizopus* sp. additional measurements, at 16 h, were performed.

#### 2.4.1. Confrontation assay (CY)

A dual screening assay in which the mold target is confronted with each yeast strain (CY assay) was used to investigate the potential inhibitory effect, mainly resulting from diffusible compounds, of the yeast strains against the four phytopathogenic fungi. Briefly, 3 μL of seven fresh yeast cultures were equidistantly positioned at 3 cm or 1.5 cm (in the case of *Penicillium* sp.) distance from the center of a YPD agar plate (ø = 90 mm), and allowed to grow during 48 h at 25° C. Then, 3 μL of a mold spore suspension were inoculated in the center of each Petri plate and again incubated at 25°C. The radial mycelial extension of the phytopathogens in the direction of each yeast was recorded. For each mold target, and each round of assays, plates without yeast inoculation were used as external controls.

#### 2.4.2. Volatile organic compounds assay (VOCs)

All yeast strains were also screened for the potential production of volatile organic compounds (VOCs assay) with inhibitory activity against the four phytopathogenic fungi. To avoid yeast-mold target contact, Petri dishes with four compartments, containing 4 mL of YPD agar in each sector were used. In two compartments, 50 μL of pre-grown fresh yeast culture were spread plated, while in the other two, 3 μL of a mold spore suspension were inoculated in central corner of the plate. For each mold target, and each round of assays, plates without yeast inoculation were used as external controls. The plates were sealed with Parafilm^®^ to prevent the outflow of volatile compounds and incubated at 25°C. Radial mycelial extension of the phytopathogen was daily recorded.

A schematic overview of the two *in vitro* antagonistic activity assays is presented in the Supplementary Figure S1.

### 2.5. Data analysis

The inhibition of radial growth-IRG (%), which considers the radial growth-RG (mm)-of the mold target in the absence (RG*_control_*) and in the presence of a yeast strain (RG*_tested_*) at the end of the incubation period, was calculated as described by Lemos Junior et al. (2016):


$$IRG\left(\%\right)=\left(1-\frac{{RG}_{\mathrm{tested}}}{{RG}_{\mathrm{control}}}\right)*100$$

The effect of each yeast strain on mold growth profiles was also quantified considering the daily measurements of radial mycelial extension during the overall established period of incubation. The area under the mycelial extension/time curve (AUC, mm.day) was calculated applying the linear trapezoid rule using GraphPad Prism software 9.3.1 (2021 GraphPad Software, San Diego, CA, United States). The obtained data were used to determine an additional parameter to assess yeast antagonistic ability (IAC; %) which relates the AUC (mm.day) of growth of the mold target in the absence (AUC*_control_*) and in the presence of a yeast strain (AUC*_tested_*) during the incubation period, calculated as follows:


$$IAC\left(\%\right)=\left(1-\frac{{AUC}_{\mathrm{tested}}}{{AUC}_{\mathrm{control}}}\right)*100$$

The reproducibility of antagonistic assays was assessed performing at least 20% of duplicate assays (CY and VOCs) using randomly selected yeast strains against the four targeted strains and evaluating the coefficient of variation (CV%) and respective confidence intervals obtained for the RG (mm) and AUC (mm.day) measurements.

Principal-component analysis (PCA) was carried out to represent yeast strains correlated to the inhibition of the four mold targets by diffusible and volatile compounds as response variables using JMP 11.0 software (SAS Inc., 2013).

As the data of the inhibitory activities (IRG and IAC) against the four targets by the two modes of action did not meet the assumptions of a normal distribution (Shapiro–Wilk test), prior to ANOVA analysis, data was transformed using the Aligned Rank Tool (ARTool) (Wobbrock et al., 2011). Pair-comparison of means was obtained by either *t*-test or Tukey’s procedure at *p* < 0.05, using JMP 11.0 software (SAS Inc., 2013).

The data set consisting of time-series measurements of mold growth in each dual-assay was analyzed using MiniTab software (2022 Minitab LLC). The similarity of the 397 yeast-induced mold target responses was calculated using the Pearson coefficient and UPGMA clustering. A cut-off value of 80% was applied for the definition of distinct response profile types. To facilitate comparisons among taxonomic groups (yeast genera and mold target), Pielou’s (*J*’) evenness index was calculated (Pielou, 1966) for the most prevalent genera (*n* > 20 isolates), as a quantitative estimate of the genomic and antagonistic distribution of the strains based on the number of profiles types of PCR-fingerprinting and of the mold responses. In other words, this evenness index expresses how evenly the strains are distributed among the different profiles obtained for each taxonomic group. If all yeast strains are represented in equal numbers in the obtained profiles, meaning great diversity, then *J*´ = 1, if one profile strongly dominates, meaning less diversity, *J*′ is close to zero (Zar, 1996).

## 3. Results

### 3.1. Molecular identification of the yeast collection

**Figure 1:**
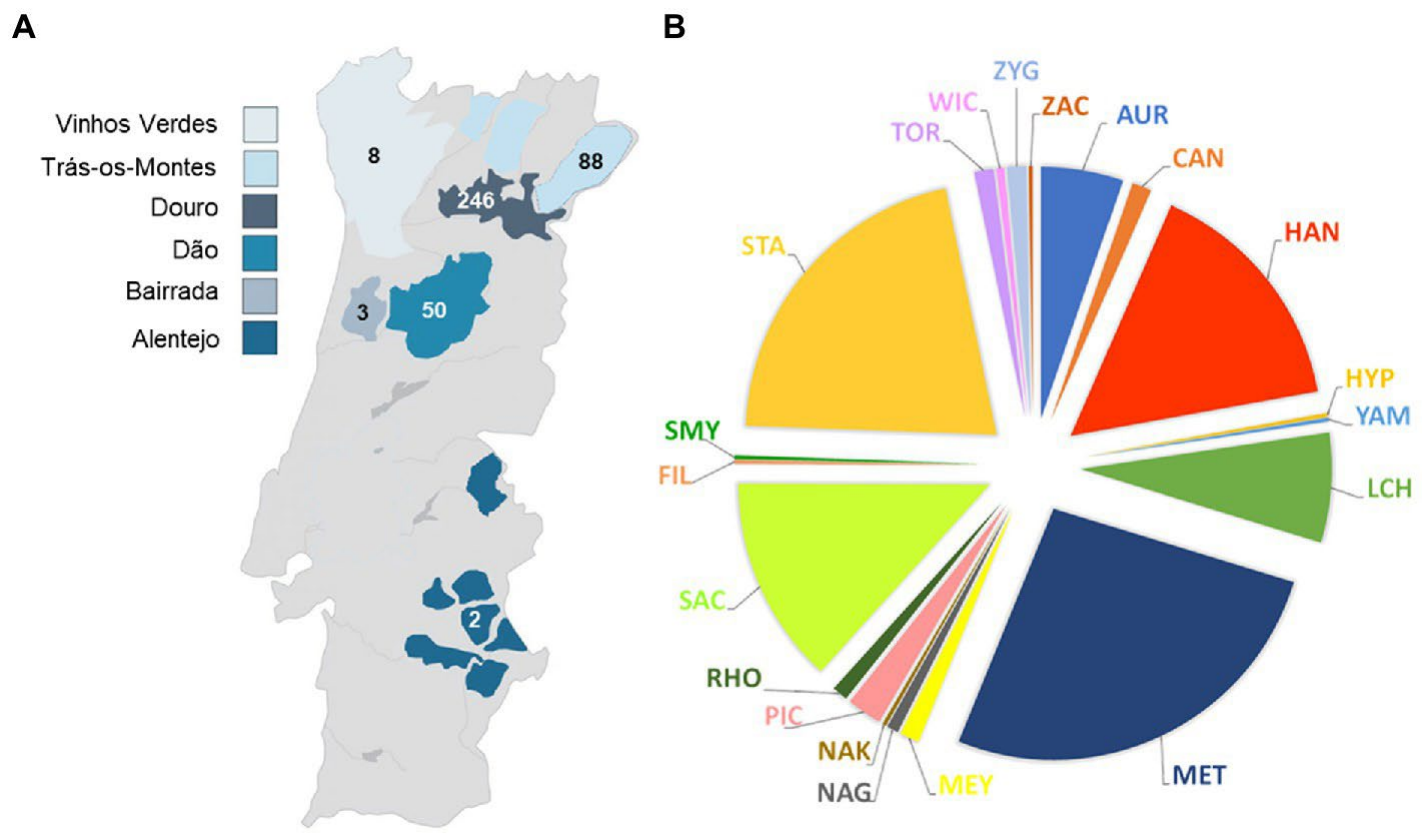
Geographical **(A)** and taxonomic distribution at the genus level **(B)** of the isolates examined in this study. The numbers of yeast isolates from the different wine regions are indicated and the colors represent the different genera. AUR, *Aureobasidium*; CAN, *Candida*; HAN, *Hanseniaspora*; HYP, *Hyphopichia*; YAM, *Yamadazyma*; LCH, *Lachancea*; MET, *Metschnikowia*; MEY, *Meyerozyma*; NAG, *Naganishia*; NAK, *Nakazawaea*; PIC, *Pichia*; RHO, *Rhodotorula*; SAC, *Saccharomyces*; FIL, *Filobasidium*; SMY, *Saccharomycodes*; STA, *Starmerella*; TOR, *Torulaspora*; WIC, *Wickerhamomyces*; ZAC, *Zygoascus*; ZYG, *Zygosaccharomyces*.

From a wider collection of autochthonous yeasts collected from different Portuguese wine regions, a subset of 397 isolates were randomly selected for this study. This set was considered to represent the regional diversity of our collection, which includes mostly isolates of samples from the Douro Demarcated Region (grapes, musts, and wines), so that most of them were expected to be originated from this region (Figure 1A). Among the set, a few isolates had been previously identified (Seixas et al., 2019); the remaining isolates, whose identity was still unknown, were herein disclosed.

Thirty-three species belonging to 20 different genera of yeasts were represented in the yeast set (Figure 1B; Supplementary Table S1). The six most prevalent genera were *Metschnikowia* (105 isolates) represented by *Met. pulcherrima*, *Starmerella* (84 isolates) represented by *Sta. bacillaris* (83) and *Sta. stellata* (1), *Hanseniaspora* (62 isolates) represented by *Han. uvarum* (48), *Han. guilliermondii* (11) and *Han. opuntiae* (3), *Saccharomyces* (53 isolates) represented by *Sac. cerevisiae*, *Lachancea* (28 isolates) represented by *L. thermotolerans* and *Aureobasidium* (21 isolates) represented by *Aur. pullulans* only. All together these genera represented approximately 89% of the isolates.

In lower numbers, we found the genera *Pichia* (9 isolates), *Candida*, *Meyerozyma, Torulaspora* and *Zygosaccharomyces* with 5 isolates each, *Rhodotorula* (4 isolates) *Naganishia* (3 isolates) and *Wickerhamomyces* (2 isolates). The genera *Filobasidium*, *Hyphopichia, Nakazawaea*, *Saccharomycodes, Yamadazyma* and *Zygoascus* were represented by a single isolate.

The sequence data of D1/D2 domains of all strains used in this study have been deposited in GenBank under the accession numbers MG832576 to MG832583, MG877743 and MG87744 (Seixas et al., 2019) and OQ304691-OQ305072.

### 3.2. Extracellular enzymatic activities of yeasts

The production of fungal cell wall-degrading enzymes is considered to be one of the important characteristics associated with the activity of biocontrol agents (Spadaro and Droby, 2016). Thus, herein, all yeasts were evaluated for extracellular enzymatic activities (amylase, glucanase, cellulase, chitinase, mannanase, protease, and pectinase).

Only 12 out of the 20 genera included strains displaying at least one enzymatic activity, and any isolate exhibited all the enzymatic activities (Table 1). Besides this intra-genus diversity, also interspecific variability was observed among strains of the same species. Six of the seven enzymatic activities tested were detected in *Aur. pullulans* strains, being the only species displaying mannanase and pectinolytic activities. In contrast, none of the *Aur. pullulans* strains exhibited glucanase activity while, notably, 21 out of 28 L. *thermotolerans* strains produced this enzyme. Protease release was found in a higher number of isolates (37), while cellulase activity was found to be the most common hydrolytic activity detected among different yeast species (7).

**Table 1:** Yeast species comprising strains with positive results in the screening performed for enzymatic activities.

Yeast species	Man	Pec	Pro	Cel	Glu	Amy	Chi
*Aureobasidium pullulans* (*n* = 21)	13	15	21	19	–	16	2
*Candida glabrata* (*n* = 4)	–	–	–	1	–	–	–
*Hanseniaspora guilliermondii* (*n* = 11)	–	–	4	–	–	–	–
*Hanseniaspora uvarum* (*n* = 48)	–	–	10	–	2	–	–
*Hyphopichia burtonii* (*n* = 1)	–	–	–	–		1	–
*Lachancea thermotolerans* (*n* = 28)	–	–	–	–	21	1	2
*Metschnikowia pulcherrima* (*n* = 105)	–	–	–	–	2	–	6
*Nakazawaea ishiwadae* (*n* = 1)	–	–	1	–	–	–	–
*Pichia kluyveri* (*n* = 2)	–	–	–	1	–	–	–
*Rhodotorula nothofagi* (*n* = 2)	–	–	–	2	–	–	–
*Saccharomyces cerevisiae* (*n* = 53)	–	–	–	1	1	–	1
*Starmerella bacillaris* (*n* = 83)	–	–	1	1	2	–	9
*Wickerhamomyces anomalus* (*n* = 2)	–	–	–	1	–	–	–
Total	13	15	37	26	28	18	20

The number of isolates tested of each species is given in parenthesis. Man, Mannanase; Pec, Pectinase; Pro, Protease; Cel, Cellulase; Glu, Glucanase; Amy, Amylase; Chi, Chitinase.

### 3.3. *In vitro* antagonistic assays

#### 3.3.1. Mold growth inhibition induced by yeasts

All 397 yeast strains were screened for their ability to inhibit four important fungal pathogens using two dual-culture growth inhibition tests. A very good intra-plate reproducibility of the assays (CY and VOCs) was found, with the average coefficient of variation for RG (mm) measurements at the end of incubation period being minor, homogeneous and similar in both types of assays (5.52% ± 1.25 and 5.73% ± 1.22% in the CY and VOCs assays, respectively).

In general, all yeasts exhibited an antagonistic phenotype, with an IRG > 5%, against at least one of the mold targets tested, through the production of diffusible (CY) and/or volatile compounds (VOCs) (Supplementary Table S1). The strains of *Pic. membranifaciens* and *R. nothofagi* exhibited the worst overall performance, not reaching 25% inhibition against any of the targets either in the CY or VOCs assays. Considering all the assays performed against all targets, the IRG (%) induced by yeasts volatile compounds production ranged from −19%, consistent with a stimulant effect by an *Aur. pullulans* strain, to 100%, corresponding to the total inhibition of *Penicillium* sp. PE3 by a *Met. pulcherrima* strain. The greatest inhibition activity against *A. niger* AN1 (81%), *B. cinerea* BO1 (94%) and *Rhizopus* sp. MU3 (94%) were also attained in the VOCs assays by a *Han. uvarum*, a *Sta. bacillaris* and a *L. thermotolerans* strain, respectively.

A stimulant effect of yeasts on mold growth was also observed in the CY assays, but only against *B. cinerea* BO1 and *Rhizopus* sp. MU3. On the other hand, the maximum inhibition levels against all targets in this assay were lower than those induced by volatile compounds: two *Han. uvarum* strains were the most effective in inhibiting the growth of *A. niger* AN1 (56%) and *Rhizopus* sp. MU3 (49%) while strains of *Sta. bacillaris* and *Sac. cerevisiae* were able to reduce 60 and 66% of the growth of *B. cinerea* BO1 and *Penicillium* sp. PE3, respectively.

**Figure 2:**
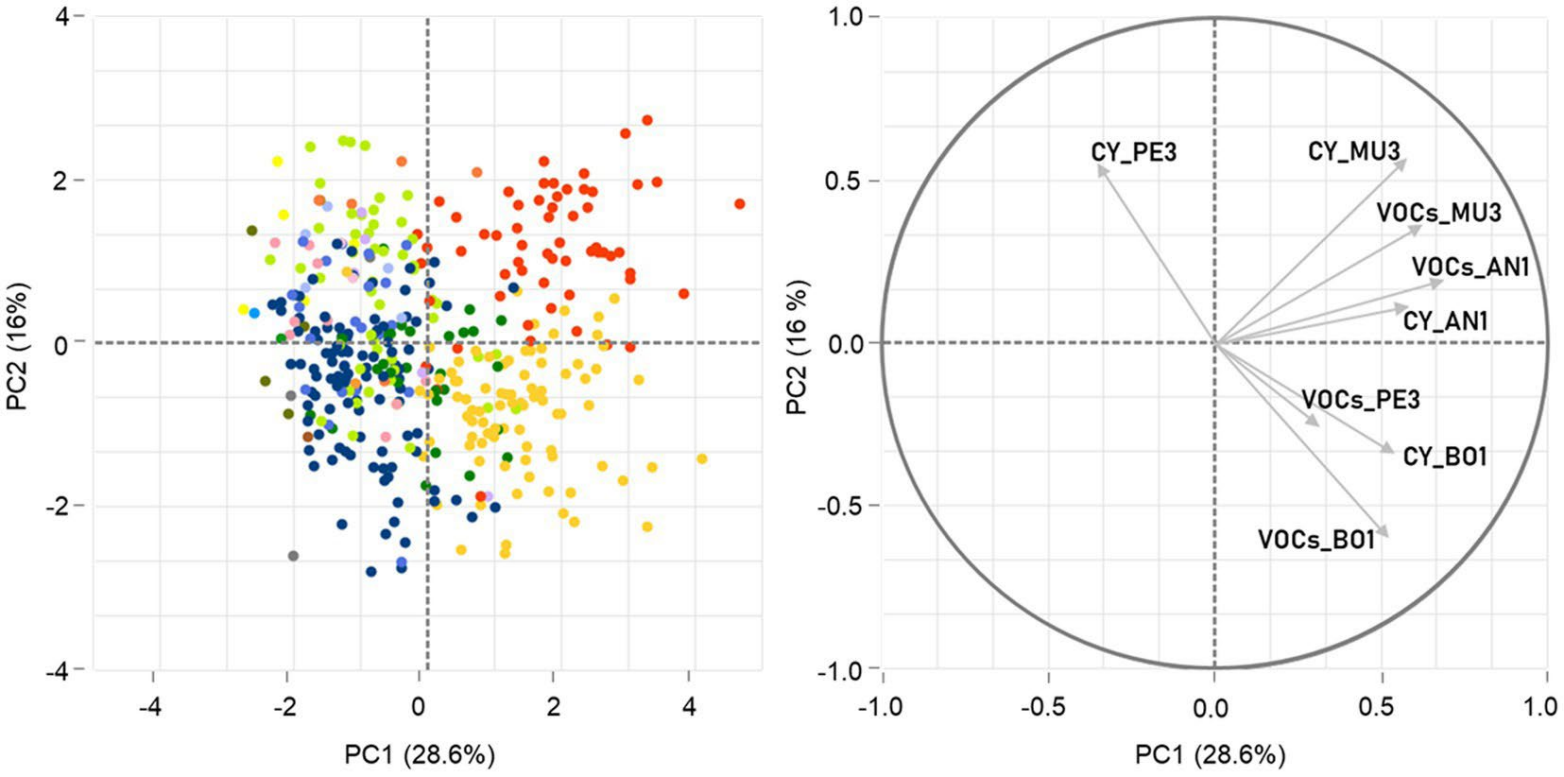
Principal component analysis (PCA) of yeast antagonistic activity against the four mold targets. The spatial representation of the 397 yeast strains according to the two first principal components (PC1 and PC2) is built on their inhibitory activity against *Aspergillus niger* AN1, *Botrytis cinerea* BO1, *Rhizopus* sp. MU3 and *Penicillium* sp. PE3, mediated by diffusible (CY) and volatile compounds (VOCs) and determined by IRG. The data points, corresponding to the yeast strains tested, were colored by genera affiliation following the color scheme used in Figure 1.

In general, the IRG (%) values obtained in the different assays for the isolates belonging to the same genus were broadly distributed and showed several outliers, as this behavior was highly variable with the target and/or the mode of action (Supplementary Table S1; Supplementary Figure S2). Indeed, the ART-ANOVA analysis showed that the antagonistic activity of this set of yeasts was significantly dependent (*p* < 0.05) on their taxonomic affiliation, the mode of action, the mold target and the interactions between all factors (Supplementary Table S2). To evaluate the overall similarity of yeast strains Principal Component Analysis (PCA) was performed, based on their combined inhibitory traits, measured in CY and VOCs assays, against the four targets (Figure 2). The results showed that only 44.6% of the total variation could be explained by the first two components of the PCA, with no clear separation of the yeast strains according to either the mold target or the mode of action. Nevertheless, a tendency for a genus-based clustering of the isolates affiliated to *Hanseniaspora* and *Starmerella,* which displayed on average significantly higher antagonistic activity (*p* < 0.05), could be noticed, with the majority of the strains grouping together away from the remaining genera according to PC1 and being separated in the first and second quadrant, respectively, according to PC2.

#### 3.3.2. Time-course mold growth responses induced by yeasts

Since that, in addition to the final growth during the incubation period, the daily extension of the mycelium was also recorded, we then sought to analyze the temporal response of each of the four mold targets to the 397 wine yeast strains. In this line, the overall time series data of *A. niger* AN1, *B. cinerea* BO1, *Rhizopus* sp. MU3 and *Penicillium* sp. PE3, obtained in the control and in the presence of each yeast strain were analyzed by hierarchical clustering with Pearson correlation coefficient and UPGMA (Supplementary Figure S3). This analysis allowed the distinction of yeasts strains based on their differential effect on the growth of each of the four mold targets, while identifying groups of strains inducing similar mold responses. For all targets, a higher number of responsive growth profiles were obtained for the CY (8–19 clusters) than for VOCs assay (4–9 clusters). Both mold target and mode of action were identified as factors driving the number and yeast genera distribution among each mold target response profile.

**Figure 3:**
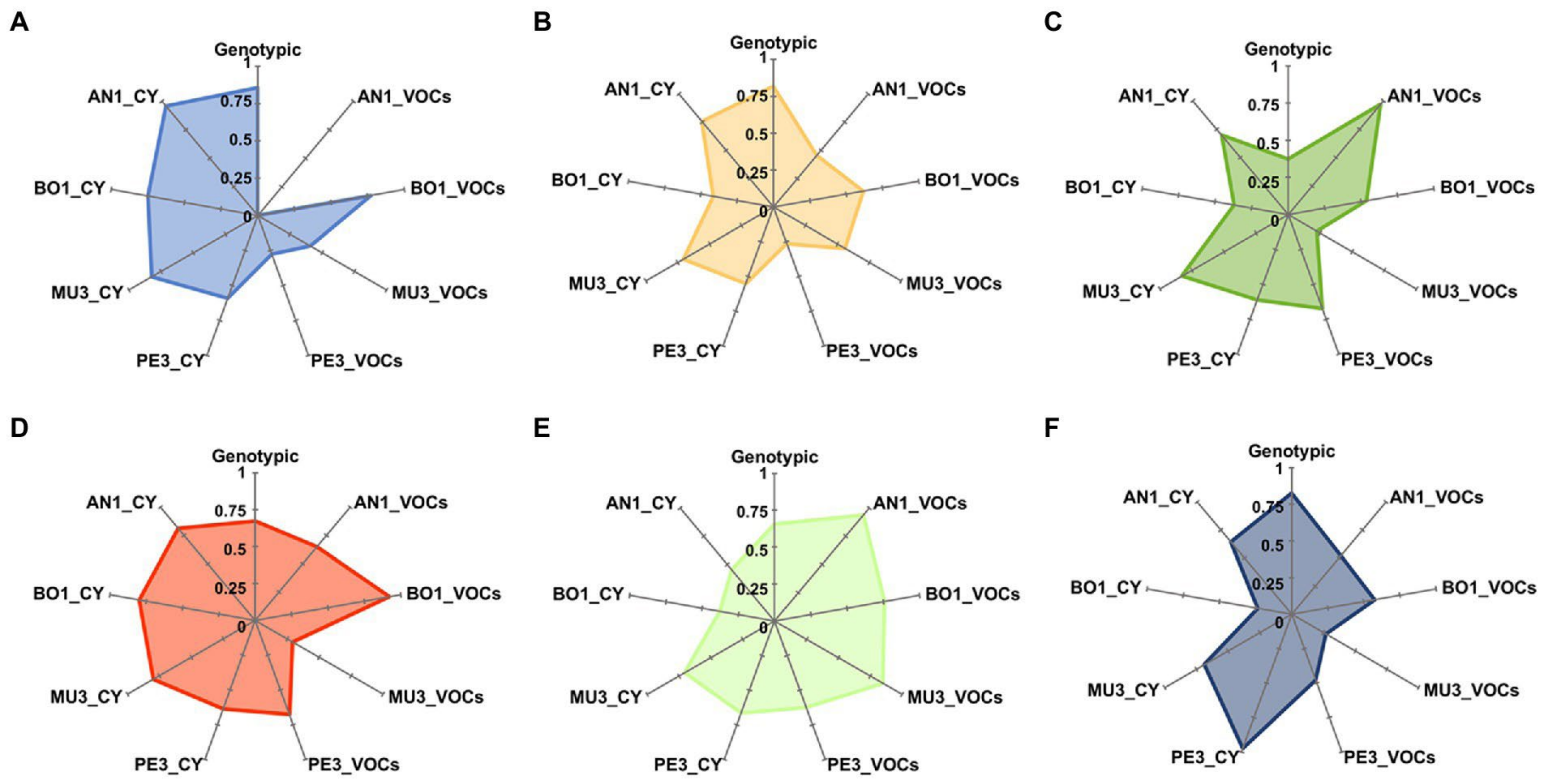
Radar plots of genotypic and antagonistic activity diversity indexes. For the most representative yeast genera, *Aureobasidium* **(A)**, *Starmerella* **(B)**, *Lachancea* **(C)**, *Hanseniaspora* **(D)**, *Saccharomyces* **(E)** and *Metschnikowia* **(F)**, Pielou’s J’ evenness index was calculated based on groups defined at 80% similarity level on dendrograms constructed, using Pearson correlation coefficient and UPGMA of the PCR fingerprinting profiles (Supplementary Figure S4) and of the mold response profiles (Supplementary Figure S3). AN1, *Aspergillus nige*r; BO1, *Botrytis cinerea*; MU3, *Rhizopus* sp.; PE3, *Penicillium* sp.; CY, diffusible compounds assay; VOCs, volatile compounds assay.

The high diversity of mold response profiles induced by the different yeast strains was further highlighted by the analysis of Pielou’s (*J*’) evenness index of diversity, calculated for the most representative genera/species (*n* > 20 isolates), using a cut-off value of 80% similarity for each dendrogram (Supplementary Figure S3) as well as in the UPGMA dendrogram of the combined (GTG)_5_ and csM13 genomic fingerprinting patterns (Supplementary Figure S4). The radar charts presented in Figure 3 illustrate the yeast inter and intra-genera diversity of induced inhibitory responses, highly dependent on both the mold target and the mode of action. Additionally, the results show that no association could be established between the genotypic diversity within a taxonomic group and the number of antagonistic responses induced by yeasts. Accordingly, despite the low genotypic diversity found among the strains of *L. thermotolerans* (*J*’ = 0.37), the 28 isolates induced a wide diversity of responses on *A. niger* AN1 (*J*’ = 0.97) and *Rhizopus* sp. MU3 (*J*’ = 0.83) in the VOCs and CY assays, respectively. Conversely, notwithstanding the high genotypic diversity found for *Aur. pullulans* (*J*’ = 0.86), the volatile compounds produced by the 21 isolates induced the same response profile on *A. niger* AN1 and highly similar ones on *Penicillium* sp. PE3 (*J*’ = 0.28).

**Figure 4:**
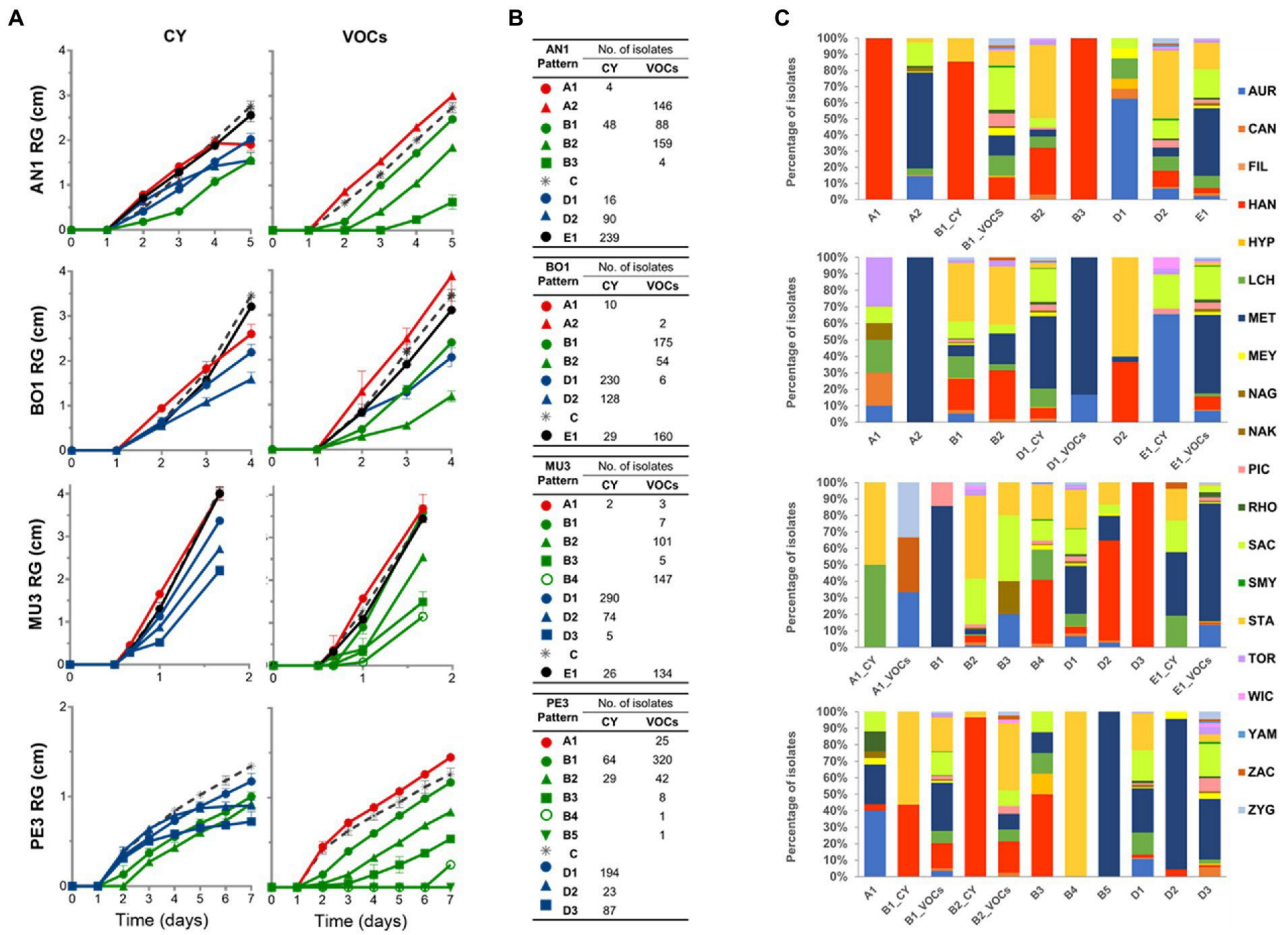
Representative growth patterns of the mold targets in response to yeast activity mediated by diffusible (CY) and volatile compounds (VOCs). Representative growth patterns of *Aspergillus niger* AN1*, Botrytis cinerea* BO1, *Rhizopus* sp. MU3 and *Penicillium* sp. PE3 in response to yeast activity were defined based on hierarchical clustering with Pearson correlation coefficient and UPGMA of time-course measurements **(A)**. Different colors were assigned to highlight the different mold growth patterns induced by yeasts: stimulatory effect (patterns A, in red); inhibition or delaying of the germination of mold spores (patterns B, in green); delayed effect on the growth rate (patterns D, in blue); no effect (pattern E, in black). For each target, mold growth pattern in the absence of yeast (control) is represented by the gray dashed line. The symbols represent mean values, with the bars indicating the standard error. The total number of isolates **(B)** and the prevalence of the yeast species **(C)** allocated to each profile are presented.

The average profiles (Supplementary Figure S3) were subsequently visually inspected and, in some cases, those displaying similar trends were merged, producing more congruent and manageable number of profiles. The ultimate distinctive growth response patterns of each mold target, as well as the number and taxonomic affiliation of the associated yeast strains, are presented in Figure 4.

The impact of yeast metabolite production could be more clearly seen in the reshaping of the growth profile of each of the four targets, with some yeast strains displaying marked effects on the germination of spores, growth rate and/or final radial mycelia extension, these being dependent on the yeast mode of action (Figure 4A). All yeast strains were found to affect the growth kinetics of *A. niger* AN1 by the production of volatile compounds and the growth of *Penicillium* sp. PE3 by either one of the type of compounds, as no response patterns clustered to the respective controls (patterns E in Figure 4A). A stimulatory effect of different yeast genera (patterns A in Figures 4A,C) on spore germination and/or on growth of all mold targets could also be observed, this being more evident in the VOCs assays for *A. niger* AN1 (146 isolates in Figure 4B). Still, the majority of the response patterns denoted antagonistic effects, at different extents, induced by yeasts against all mold targets (patterns B and D in Figure 4A).

Overall, the most prevalent response pattern prompted by yeasts, detected in the CY assays, was characterized by a decrease of mold mycelia extension rate (patterns D in Figures 4A,B). The particular average response patterns A1 and D2 of *A. niger* AN1 and D2 and D3 of *Penicillium* sp. PE3 (patterns in Figure 4A) should be highlighted as they denote yeast-induced effects characterized by a significant reduction or even the early complete arrest of mycelial extension, after an initial regular mold growth.

Volatile compounds had a more marked effect on the timing of the onset of spore germination, inducing more or less longer lag growth phases of all targets, except *B. cinerea* BO1 (patterns B in Figures 4A,B). These plateaus are disregarded by the commonly used IRG (%) metric that only considers the final RG (mm) and thus may underestimate the categorization of yeast antagonistic potential. Indeed, while the IRG (%) value seems to adequately reflect the antagonistic activity of yeasts when the mycelial extension rate is steady across the incubation period, this may not be the case when either the onset of spore germination or mycelial extension rate is differentially affected. An illustrative example is the similar average IRG (%) of the yeast strains assigned to patterns B1 and D2 for *A. niger* AN1 or assigned to patterns B2 and D2 for *Penicillium* sp. PE3, despite the marked differences between the mold growth patterns.

Collectively, those observations prompt us to propose a new metric (IAC, %), with a higher discriminating power, for the categorization of yeasts based on the determination of the area under the curves (AUC, mm.day) of mold growth in the absence and in the presence of the different yeast strains, during the incubation period (Supplementary Table S1). As seen for RG (mm) measurements the average variance of the determined AUC (mm.day) values was low and homogeneous in both assays, although higher in the VOCs assay (8.67% ± 1.95%) than in the CY (4.68% ± 0.6%). The two metrics were highly correlated, particularly in the VOCs assays ( > 90%) irrespective of the mold target (Supplementary Figure S5). With the exception of *B. cinerea* BO1, lower correlations were found between the metrics determined for the CY assays, this being associated with the higher number of yeast strains inducing more pronounced variations or the abrupt arrest in the mycelial extension rate during the incubation period in these assays (Figures 4A,B).

Principal component analysis was also performed to assess the distribution of the 397 yeast strains based on IAC (%) (Supplementary Figure S5). Although the amount of total variation explained (46.6%) was not markedly higher than that obtained using the IRG (%) metric (Figure 2), the use of the IAC (%) values led to a better genus-based distribution of the yeast strains built on the antagonistic activity (PC1) and a higher effectiveness of PC2 in distinguishing the mode of action of yeast strains within each genus, as demonstrated by the direction and magnitude of the respective PCA score plots. These results are more in agreement with the significant differences found in ART-ANOVA analysis of the inhibitory activity among genera and within each genus according to the different types of metabolites produced by yeasts (Supplementary Table S3).

#### 3.3.3. Diversity of yeasts with antagonistic activity against the mold targets

**Figure 5:**
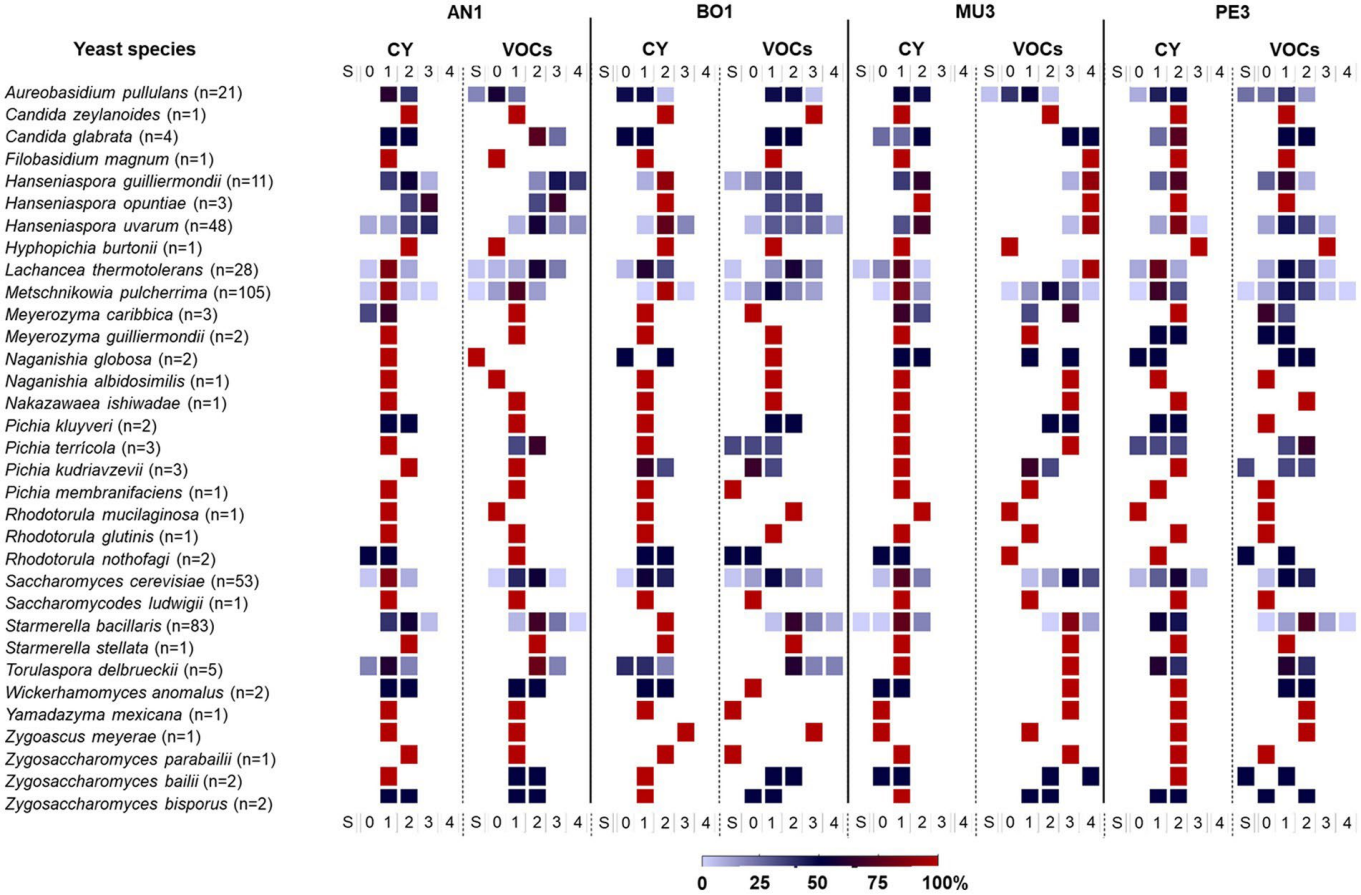
Frequency distribution of yeast strains for each species based on their antagonistic activity against the four mold targets. Classes of yeast strains were defined based on IAC values: class 0 (no effect: IAC < 5%), class 1 (weak effect: 5% ≤ IAC < 25%), class 2 (moderate effect: 25% ≤ IAC < 50%), class 3 (strong effect: 50% ≤ IAC < 75%) and class 4 (very strong effect: IAC ≥75%). Strains with stimulatory effect of mold growth were included in class S. The color gradient refers to the percentage of strains in each class. AN1 – *Aspergillus niger*; BO1 – *Botrytis cinerea*; MU3 – *Rhizopus* sp.; PE3 – *Penicillium* sp.; CY – diffusible compounds assay; VOCs – volatile compounds assay. The number of isolates tested of each species is given in parenthesis.

For a biological interpretation of the data, the yeast strains were classified in different categories based on their inhibitory activity using the IAC (%) metric: class 0 (no effect: -5 < IAC < 5%), class 1 (weak effect: 5% ≤ IAC < 25%), class 2 (moderate effect: 25% ≤ IAC < 50%), class 3 (strong effect: 50% ≤ IAC < 75%) and class 4 (very strong effect: IAC ≥ 75%). Strains with stimulatory activity, identified by a negative IAC (< −5%) effect of mold growth were included in class S. Figure 5 summarizes the distribution of the yeast strains for each species on those classes. In accordance with the above description of yeast-induced diversity of mold responses (Figure 3), the distribution of the IAC values amongs yeast strains was found to be heterogeneous, and none of the yeast genus/species represented by more than one strain comprised only one class of strains against all targets. Furthermore, the data presented in Figure 5, underscores the variability in the biocontrol potential found among yeasts of the same taxonomic group. For example, the 105 Met. *pulcherrima* strains were considered for all the six classes, from S through 4, according to their VOCs-induced effect on *Penicillium* sp. while the three strains of *Han. opuntiae* tested were placed in classes 1,2, and 3, based to their VOCs-induced antagonistic activity (22, 44, and 59%, respectively) against *B. cinerea* BO1.

In order to identify the most promising candidates to be used as biocontrol agents we restrained our subsequent analysis to those isolates exhibiting a more prominent inhibitory activity by selecting strains only included in classes 3–4 by both type of assays (CY and VOCs). Interestingly, no isolate was found in those classes for *Rhizopus* sp. MU3 in the CY assay and thus a less stringent criterion was applied, and isolates included in class 2 for this target were considered instead in the following analysis.

**Figure 6:**
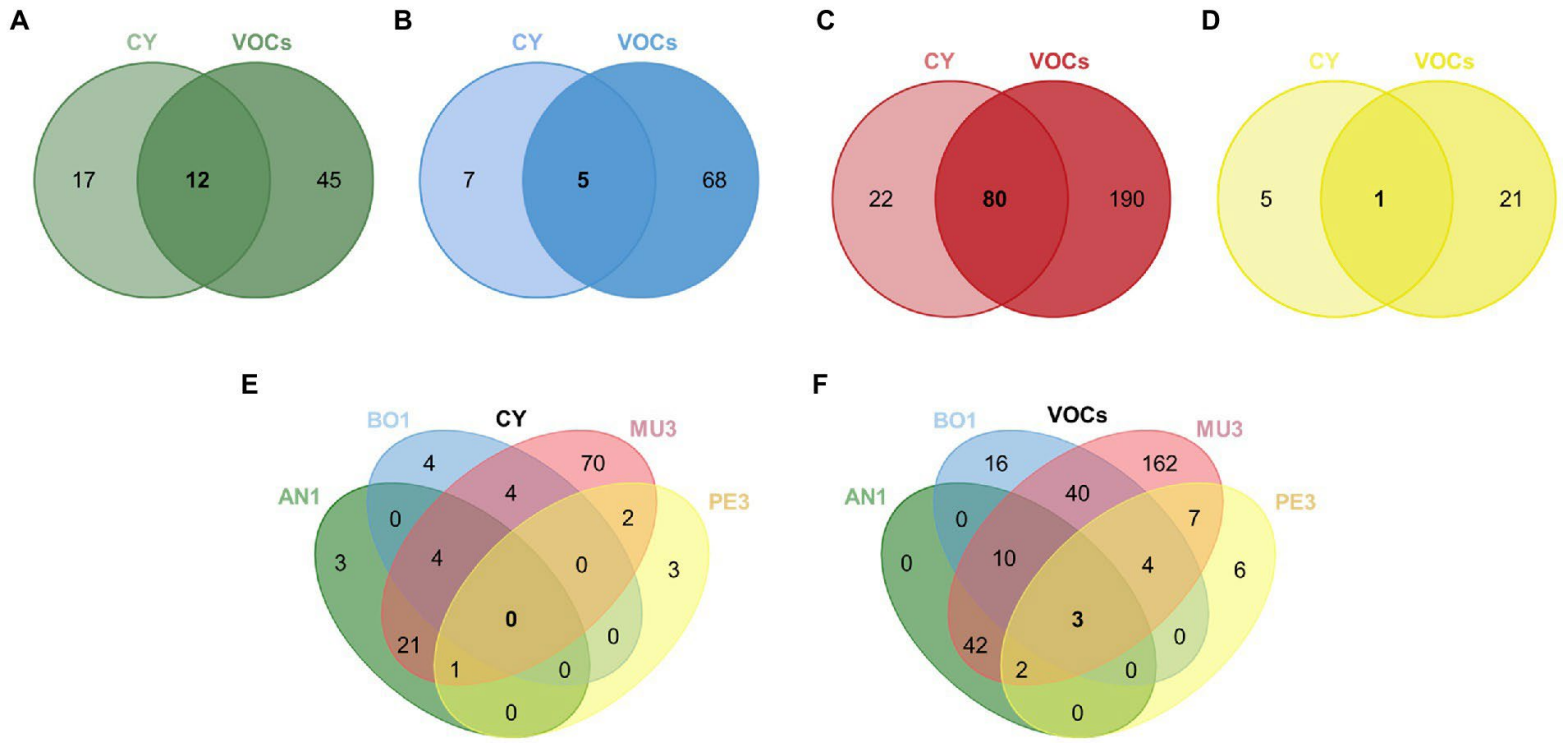
Venn diagrams illustrating the number of yeast strains with inhibitory activity against each target individually, *Aspergillus nige*r AN1 **(A)**, *Botrytis cinerea* BO1 **(B)**, *Rhizopus* sp. MU3 **(C)** and *Penicillium* sp. PE3 **(D)**, or against all targets **(E,F)** mediated by diffusible (CY) and volatile compounds (VOCs). The number of strains classified on class 3 (strong effect: 50% ≤ IAC < 75%) or class 4 (very strong effect: IAC ≥ 75%) for each mold target were included, except for MU3 in the CY assay, where only strains classified on class 2 were included (moderate effect: 25% ≤ IAC < 50%).

In a first approach we aimed to identify the most effective strains against each mold target, irrespective of the mode of action. The Venn diagrams presented in Figures 6A–D present the number of shared strains included in these categories for each target. Irrespective of the type of assay, the percentage of yeast isolates with strong and or very strong antifungal activity was the highest against *Rhizopus* sp. MU3 (68%) followed by *B. cinerea* BO1 (20%), *A. niger* AN1(19%) and *Penicillium* sp. PE3 (7%).

Twelve isolates were found to be highly effective by both modes of action against *A. niger* AN1 and comprise strains of *Sta. bacillaris* (1), *Han. uvarum* (9) and *Han. opuntiae* (2) (Figure 6A). These yeasts were all associated with the response patterns B1_CY and B2, except one *Han. uvarum* that was assigned to the pattern B3 (Figure 4). The average IAC in both assays ranged from 71 to 51.8% induced by two *Han. uvarum* strains.

Three isolates of *Han. uvarum*, one of *Met. pulcherrima* and the only representative of *Zygoascus meyerae* showed a strong inhibitory activity against *B. cinerea* BO1 by both modes of action (Figure 6B). The latter displayed the highest average IAC (65.7%) and was part of the strains inducing a D1 and B2 response patterns in *B. cinerea* (Figure 4).

As for *Rhizopus* sp. MU3, 80 isolates, belonging to eight different genera, were considered as potentially interesting biocontrol strains with an average IAC (%) in both assays ranging from 39.1% by one *Sac. cerevisiae* strain, assigned to patterns D1 and B2 (Figure 4), to 71.8% by one *Han. uvarum* allocated to patterns D3 and B4 (Figure 3).

The single strong inhibitor of *Penicillium* sp. PE3, with an average IAC of 58.3%, was the only representative of *Hyphopichia burtonii* in our set of yeasts. This yeast was included in the group of strains inducing a D3 and B3 response patterns in this mold target (Figure 4), being able to completely inhibit *Penicillium* sp. PE3 proliferation since day 5 in the CY assay.

Additionally, the strains affiliated to *Pic. terricola* (2) and *Mey. guilliermondii* (2), which inhibited *Penicillium* sp. PE3 growth since day 3 until de end of the experiment (patterns D3, Figures 4A,B), and the strains of *Aur. pullulans* (2), *Can. glabrata*, *Han. uvarum*, *Hyp. burtoni, L. thermotolerans*, which effectively led to an early arrest of *A. niger* AN1 growth (patterns A1 and D2, Figures 4A,B), warrants them being considered in a future selection program due their remarkable effects.

We then aimed to identify strains with broad spectrum of antagonistic activity (Figures 6E–F). No yeast strain was found to be highly effective against all four mold targets through the production of diffusible compounds. Nevertheless, we have identified five *Han. uvarum* strains that are strong inhibitors of three of the four targets. While four strains were effective against *A. niger* AN1*, B. cinerea* BO1 and *Rhizopus* sp. MU3, one other strain was effective against *A. niger* AN1*, Rhizopus* sp. MU3 and *Penicillium* sp. PE3. On the other hand, three strains affiliated to different species – *H. uvarum, L. thermotolerans* and *Sta. bacillaris* were identified as having strong or very strong antagonistic activity through the production volatile compounds against all the targets tested, with average IAC (%) values of 73, 67, and 68%, respectively.

## 4. Discussion

*Vitis vinifera* L. grapevine is one of the most cultivated (in an estimated surface area of 7.3 million hectares) and valued fruit crops, given the many applications of grape production, from its fresh consumption (table grapes and raisins) to its use in the production of juices and wines (FAO-OIV, 2016; OIV, 2022). Accordingly, a great wealth of technology investment is devoted to control the development of fungal rots that may occur during grape maturation and postharvest, and reduce the quality and safety of the final product. These phytopathogenic fungi include *B. cinerea,* which is responsible for severe gray rot, during pre- and post- harvest (Kassemeyer, 2017); several species belonging to *Aspergillus* and *Penicillium,* that besides being responsible for black and green/blue rots, respectively, are recognized as producers of mycotoxins that can cause a risk for human health (Serra et al., 2005); and *Rhizopus* spp. which are mainly known as post-harvest disease causing agents that can develop even at cool storage conditions (Kassemeyer, 2017).

Presently, there is an increasing demand for more sustainable and eco-friendly practices in the agricultural ecosystems aiming the replacement, or at least the reduction, of the chemical products that are currently being used (Wilson and Wisniewski, 1989; Pertot et al., 2017b). A considerable number of reports have demonstrated the antagonistic behavior of several strains of different yeast species against phytopathogenic fungi, pointing out their potential for biocontrol applications (Zhang et al., 2020; Di Canito et al., 2021). Despite the demonstrated efficiency, the number of yeasts available at the market for using as biofungicides in grapes against phytopathogenic fungi in the pre- and/or postharvest stages remains limited (Palmieri et al., 2022). According to the data available on EU pesticide database, accessed on 13 January 2023, there are only two yeast-based products being commercialized or submitted for approval, with different active substances (yeasts), Julieta^®^ (*Sac. cerevisiae* LAS02) and Shemer^®^ (*Met. fructicola* NRRL Y-27328), both targeting only *B. cinerea*. Thus, the quest for new yeasts with reliable biocontrol prospects against grape phytopathogenic fungi continues.

In this study 397 wine yeast strains were tested for their biocontrol potential against four major grapes fungal pathogens, *A. niger*, *B. cinerea*, *Rhizopus* sp. and *Penicillium* sp. This set of yeast strains, isolated from grapes/grape-juices and spontaneous grape-juice fermentations derived from different Portuguese wine-regions, was found to be highly diverse in their taxonomic groups, encompasses mostly non-*Saccharomyces* species (approximately 87%) and 53 *Sac. cerevisiae* strains and thus represents the broad biodiversity common in the targeted niche (Fleet and Heard, 1993; Bokulich et al., 2014; Pinto et al., 2015). The exploration of these epiphytic yeasts as biocontrol agents arises as a valuable option as they have the advantage of not disrupting the local ecological makeup. In addition, while being in their natural ecosystem they can evolve better and easily adjust to the associated environmental conditions, thus being more prone to demonstrate effective and consistent biocontrol activity against grapes phytopathogens (Parafati et al., 2015; Pretscher et al., 2018; Pereyra et al., 2021). In fact, most of the species represented in our collection include previously reported antagonists of grape phytopathogenic fungi (Suzzi et al., 1995; Bleve et al., 2006; Raspor et al., 2010; Nally et al., 2012; Pantelides et al., 2015; Lemos Junior et al., 2016; Cordero-Bueso et al., 2017; Pretscher et al., 2018; Reyes-bravo et al., 2019) while the antagonistic potential of *Nakazawaea ishiwadae* and *Yamadazyma mexicana* are herein reported for the first time.

However, the lack of standardized protocols for *in vitro* dual culture assays is a major drawback for the direct comparison of the results obtained in the different studies and the accomplishment of definitive classification of antagonistic yeasts. Indeed, the level of yeast inhibition has been distinctly evaluated, either by arbitrary scales (Suzzi et al., 1995; Bleve et al., 2006), inhibition halos (Nally et al., 2012; Cordero-Bueso et al., 2017; Reyes-bravo et al., 2019) or percentages (Raspor et al., 2010; Pantelides et al., 2015; Lemos Junior et al., 2016; Pretscher et al., 2018; Reyes-bravo et al., 2019)(Lemos Junior et al., 2016; Reyes-bravo et al., 2019) obtained by comparing the mold colony sizes in the presence of the yeasts with that for its single culture at a defined end time point. In addition, the use of a panoply of distinct methodologies related with the yeast inoculation technique (incorporation, spot, streak or spread plating), type of mold inoculum (spore suspension or mycelial plug), nature of interaction (direct or indirect contact), period of incubation and the culture medium used, introduce even more entropy in the data comparison.

In this work, yeast strains with strong inhibitory activity ( > 50%) against all the phytopathogenic fungi tested were found. Overall, the strains affiliated to species of *Hanseniaspora*, *Lachancea* and *Starmerella* tested in our study were the most effective against *A. niger*, *B. cinerea* and *Rhizopus* sp. Previous studies have already reported wine strains of *Han. uvarum* and *Sta. bacillaris* with strong inhibitory activity against *Aspergillus* spp. and *B. cinerea* (Lemos Junior et al., 2016; Cordero-Bueso et al., 2017; Fernandez-San Millan et al., 2021) and of *L. thermotolerans* against *Aspergillus* spp. (Bleve et al., 2006). Although *L. thermotolerans* have already been tested against *B. cinerea* (Nally et al., 2012; Fernandez-San Millan et al., 2021), our report is the first to demonstrate the antagonism of a wine yeast strain of this species against this grape phytopathogen. Strains of *Sac. cerevisiae*, *Sta. bacillaris* and the single strain of *Hyp. burtonii* were found as strong inhibitors of *Penicillium* sp. growth. While wine strains of *Sac. cerevisiae* and *Sta. bacillaris* have already shown antagonistic behavior against *P. expansum* (Cordero-Bueso et al., 2017; Fernandez-San Millan et al., 2021) the biocontrol potential of *Hyp. burtonii* against *Penicillium* spp. is herein reported for the first time. However, despite being poorly studied, this species has previously shown biocontrol potential against other phytopathogenic fungal targets such as *Alternaria alternata*, *Aspergillus niger* and *B. cinerea*, either by strains isolated from green coffee beans (Poitevin et al., 2020) or grape-must (Maluleke et al., 2022).

Biocontrol activity of yeasts by direct mold antagonism is mainly mediated by the competition for nutrients and space, secretion of non-volatile compounds (including cell wall degrading enzymes) and production of volatile compounds (Freimoser et al., 2019). In this work, besides the search for secreted hydrolytic enzymes, the yeast antagonistic activity was evaluated using two designs of dual-culture assays, directed to either the production of diffusible (CY assay) or volatile compounds (VOCs assay). The evaluation of the different putative modes of action already points toward different prospective applications, in pre- and/or postharvest grapes. Accordingly, the prior inoculation of fast colonizer strains aided by production of antagonistic diffusible compounds (CY assays), could be envisaged for both pre- and postharvest applications, as a preventive intervention to control development. Yet the safety of the direct application of such biocontrol agents on the grape surface must be assured as there are reported cases of infections caused by yeasts in humans (Nally et al., 2013). On the other hand volatile compounds (VOCs assays), which may have a limited activity in the open field (Song and Ryu, 2013), but present reduced hazard for both environment and human beings (Tilocca et al., 2020) would be more likely indicated for post-harvest applications, under more controlled environments.

In addition, the original time-course mold growth monitoring approach, performed in the present study, allowed the detection of a high diversity of mold growth effects induced by yeasts. Indeed, it has been demonstrated that the comparison based on time series analysis enables a more thorough and objective comparison of the influence of environmental conditions on mold growth dynamics (De Ligne et al., 2019). All the aforementioned studies that evaluated the antagonistic effect of yeasts on phytopathogenic mold growth, neglect time, measuring mold growth inhibition after a predetermined number of days. Additionally, since the yeast effects on the inhibition of spore germination may differ from those involved in the inhibition of mycelial extension, the use of spore suspensions coupled with daily monitoring of mold growth allowed us to identify potentially effective new strains that could not be revealed by using mycelial plugs. Taken together, our approach further uncovered important yeast-mediated effects throughout the experiments besides the commonly measure of reduction of mold growth, including the inhibition or delay of spore germination and the complete arrest of mycelial extension, and even their stimulation at different mold growth stages, this being highly dependent on yeast species/genus, mode of action and mold target.

Herein we uncover a remarkable intra and interspecific diversity in the yeast inhibition levels against mold targets, which is in line with previous works (Suzzi et al., 1995; Raspor et al., 2010; Lemos Junior et al., 2016). Besides, our approach also emphasizes the wide range of yeast-induced antagonistic profiles across different yeast species and strains. Yet, no association could be established between the genotypic diversity within a species and the respective diversity of antagonistic responses, nor with the intensity of the inhibition induced by strains, demonstrating that taxonomic affiliation is not a reliable predictor for biocontrol potential.

The diversity of yeast-mediated mold growth responses led us to propose another parameter, IAC (%), based on the area under the mycelial extension/time curve (AUC; mm.s), which integrates the different effects over time. The AUC measure is also preferred in other fields of study such as pharmacology where, for example, it is used to accurately estimate the extent of a body’s exposure to a particular drug over time (Scheff et al., 2011). The use of IAC (%) allows ascribing greater prominence to antagonists that inhibit spore germination and that would be more suitable for preventive treatments, as their associated AUCs would be low. However, the IAC (%) underestimates the inhibitory effect of the strains that lead to the early cessation of mold growth, and would likely be excluded during the selection process, as the AUC (mm.day) will be accounted throughout. This highlights the importance of analyzing the mold growth profiles as an independent step, yet complementary to the calculated inhibition parameters, IRG (%) and IAC (%), during a selection program.

Overall, the response patterns prompted by yeast in the CY assays correspond to a decrease of mold mycelia extension rate, that ultimately led to the early complete arrest of mycelial extension, after a regular initial growth. Our CY assay setup does not allow distinguishing the production of diffusible metabolites (antibiosis), or competition for nutrients, or the secretion of extracellular enzymes as the underlying mechanism of inhibition (Freimoser et al., 2019). However according to our results, we could not find a direct link between the secretion of glucanases, chitinases, proteases and mannanases, that may degrade the cell wall constituents of the tested molds, and the antagonism observed in the CY assays. Indeed, our screening for hydrolytic enzymatic activities uncovered intra-genus and several intraspecific differences within the yeast isolates, with *Aur. pullulans* strains exhibiting the highest diversity of lytic enzymes released, in line with the recognized ability of this species (Bozoudi and Tsaltas, 2018). On the other hand, glucanase activity was highly frequent in our *L. thermotolerans* strains, a feature that has been previously identified for this species (Romo-Sánchez et al., 2010), although it is considered rare (Vicente et al., 2021). Nevertheless, the role of these hydrolytic enzymes on the inhibitory activity of these species was unclear, as no enhanced antagonism was detected in CY assays.

In the CY assays, a strong deceleration, or even the arrest, of mycelial extension of mold growth, induced mainly by *Met. pulcherrima* and *Sta. bacillaris* was detected against the most phylogenetically closer targets, *A. niger* and *Penicillium* sp. Also, a delay in spore germination of these mold targets by strains of *Hanseniaspora* spp. and *Sta. bacillaris* was found. These observations suggest the involvement of diffusible compounds on mold inhibition, with the distinct effects being dependent on their toxicity, and rates of production and diffusion (Swadling and Jeffries, 1996). In practice, yeasts strains with marked effects on mold mycelial extension, may be also important as protective agents to reduce disease severity and spread to other fruit clusters. Regardless, depending on the concentration of the active compound(s), these inhibitory effects are amenable to be optimized to achieve maximum effectiveness in practical applications.

The production of volatile compounds appeared to be a major mechanism of yeast-mediated inhibition of the four mold targets, with maximum IACs (%) surpassing 90%. Alcohols and their respective esters, have been associated with the antagonistic activity of yeasts against phytopathogenic fungi (Tilocca et al., 2020). The most reported inhibitory volatile compounds produced by yeasts include 2-phenylethanol and its respective ester 2-phenylethyl acetate and 1,3,5,7-cyclooctatetraene and 3-methyl-1-butanol, 2-ethyl-hexanol and ethyl acetate (Masoud et al., 2005; Huang et al., 2011, 2012; Hua et al., 2014; Di Francesco et al., 2015; Farbo et al., 2018; Oro et al., 2018). Wine yeasts have been described as large producers of several of these volatile compounds, mainly in the context of grape-juice fermentation where they are interesting for the development and complexity of wine aroma (reviewed in Petruzzi et al., 2017; Liu et al., 2022). A formulation of a product with a bi-functional role of application, as a biocontrol and bioflavoring agent could then be foreseen for the strains of these species that presented the best inhibitory performances in this work, as proposed by Lemos Junior et al. (2016).

A relevant effect induced on the mold targets by volatile compounds was the delay on spore germination, detected in *A. niger*, *Rhizopus* sp. and *Penicillium* sp. These compounds are known to be used by fungi as chemical signals to control physiological processes such as nutrient acquisition, sporulation, sexual development and spore germination (Leeder et al., 2011; Bennett et al., 2012). It is known that when too many spores exist in proximity, fungi may produce volatile compounds to act as self-inhibitors of spore germination (Bitas et al., 2013). The volatile compounds produced by the yeast strains may have a similar chemical constitution and be perceived as self-inhibitors or in turn, other compounds with different chemical structure may be produced that disturb mold chemical signaling. In the present study, *Rhizopus* sp. has been shown to be the most susceptible to the volatile compounds produced, with 68% of yeast strains being strong or very strong inhibitors, particularly strains of *Han. uvarum, L. thermotolerans*, *Sac. cerevisiae* and *Sta. bacillaris*. This result suggests that *Rhizopus* sp. sporangiospores might be more sensitive than the conidia of the ascomyceteous fungi, especially those of *B. cinerea*, to the volatile compounds produced by the wine yeasts strains. A discrepant sensitivity between spores of *B. cinerea* and *Mucor racemosus* has been reported by Catskx et al. (1975) that studied the effects of volatile metabolites released by swelling seeds on spore germination of five genera of fungi.

Strikingly, a *Met. pulcherrima* strain was found in this work to completely inhibit the spore germination of *Penicillium* sp. through the production of volatile compounds. The antagonistic activity of *Met. pulcherrima* strains has been for long reported and is mainly associated with the iron depletion and diffusion of the pulcherrimin pigment (Sipiczki, 2006). Our findings may therefore be useful to further expand its range of applications. Several strains of other species, mainly of *Han. uvarum* and *Sta. bacillaris* showed a temporary inhibition of spore germination of all the mold targets, except *B. cinerea*. From the practical point of view, complete inhibition of spore germination is always the most desirable as a treatment, preventing fungal proliferation and associated diseases in fruits. These strains could be useful to achieve grapes protection during the timeframes more crucial to target pathogen proliferation in the vineyard. At postharvest, they could be as well advantageous during transportation and storage, to extend the fruit shelf-life.

## 5. Conclusion

The time-course analysis performed in this study provided a robust exploitation of the biocontrol potential of a large set of wine yeasts, enabling the identification of yeast effects on mold targets at different growth stages. The wide diversity of inhibitory effects found, highlighted the importance of establishing target-and application-oriented protocols for yeast strain selection. Herein, a catalog of potential biocontrol agents, including several with a large target-spectrum of activity and versatility of mode of action was established. Particularly, strains of *Han. uvarum*, *L. thermotolerans*, *Met. pulcherrima* and *Sta. bacillaris* stood out as the best candidates for application either in pre- or post-harvest grapes, being very interesting for further research. Yeast consortia, combining yeasts with distinct modes of action and effects on spore germination and mold growth, could be a promising strategy in the formulation of new ecosystem-based tools as environmentally friendly alternatives to chemical fungicides, supporting the development of a more sustainable vitiviniculture.

## Data availability statement

The datasets presented in this study can be found in online repositories. The names of the repository/repositories and accession number(s) can be found in the article/Supplementary material.

## Author contributions

AM-F, FC, and MT were responsible for funding acquisition, AM-F and RT conceived and designed the experiments. ME, PL, and JS performed the experiments. ME prepared the original draft. ME, PL, FC, RT, and AM-F organized and analyzed the data. ME, RT, and AM-F wrote the manuscript. AM-F supervised the study. All authors contributed to the article and approved the submitted version.

## Funding

This work was financially supported by ABCyeasts project no. NORTE-01-0247-FEDER-039793, co-financed by FEDER through NORTE 2020. ME is a recipient of a PhD grant with the reference PD/BD/150587/2020 from the FCT Doctoral Program in Applied and Environmental Microbiology (DP_AEM).

## Conflict of interest

FC and MT was employed by Proenol. 

The remaining authors declare that the research was conducted in the absence of any commercial or financial relationships that could be construed as a potential conflict of interest.

## Correction note

A correction has been made to this article. Details can be found at: 10.3389/fmicb.2026.1817348.

## Publisher’s note

All claims expressed in this article are solely those of the authors and do not necessarily represent those of their affiliated organizations, or those of the publisher, the editors and the reviewers. Any product that may be evaluated in this article, or claim that may be made by its manufacturer, is not guaranteed or endorsed by the publisher.

## References

[ref1] Asfamawi K. K. Noraini S. Darah I. (2013). Isolation, screening and identification of mannanase producing microorganisms. J. Trop. Agric. Fd. Sci. 1, 169–177.

[ref2] Barata A. Malfeito-Ferreira M. Loureiro V. (2012). The microbial ecology of wine grape berries. Int. J. Food Microbiol. 153, 243–259. doi: 10.1016/j.ijfoodmicro.2011.11.025, 22189021

[ref3] Barbosa C. Lage P. Esteves M. Chambel L. Mendes-Faia A. Mendes-Ferreira A. (2018). Molecular and phenotypic characterization of *Metschnikowia pulcherrima* strains from Douro wine region. Fermentation 4:8. doi: 10.3390/fermentation4010008

[ref4] Bélanger R. R. Labbé C. Lefebvre F. Teichmann B. (2012). Mode of action of biocontrol agents: all that glitters is not gold. Can. J. Plant Pathol. 34, 469–478. doi: 10.1080/07060661.2012.726649

[ref5] Bennett J. W. B. Hung R. H. Lee S. L. Padhi S. P. (2012). “Fungal and bacterial volatile organic compounds: an overview and their role as ecological signaling agents” in The Mycota IX Fungal Interactions. ed. B. Hock (Berlin Heidelberg: Springer-Verlag), 373–393.

[ref6] Bitas V. Kim H. Bennett J. W. Kang S. (2013). Sniffing on microbes: diverse roles of microbial volatile organic compounds in plant health. Mol. Plant-Microbe Interact. 26, 835–843. doi: 10.1094/MPMI-10-12-0249-CR, 23581824

[ref7] Bleve G. Grieco F. Cozzi G. Logrieco A. Visconti A. (2006). Isolation of *Epiphytic yeasts* with potential for biocontrol of *Aspergillus carbonarius* and *Aspergillus niger* on grape. Int. J. Food Microbiol. 108, 204–209. doi: 10.1016/j.ijfoodmicro.2005.12.004, 16443300

[ref8] Bokulich N. A. Thorngate J. H. Richardson P. M. Mills D. A. (2014). Microbial biogeography of wine grapes is conditioned by cultivar, vintage, and climate. Proc. Natl. Acad. Sci. 111, E139–E148. doi: 10.1073/pnas.1317377110, 24277822 PMC3890796

[ref9] Bozoudi D. Tsaltas D. (2018). The multiple and versatile roles of *Aureobasidium pullulans* in the vitivinicultural sector. Fermentation 4, 4–S5. doi: 10.3390/fermentation4040085

[ref10] Catskx V. Afifi A. F. Vancura V. (1975). The effect of volatile and gaseous metabolites of swelling seeds on germination of fungal spores. Folia Microbiol. 20, 152–156. doi: 10.1007/BF02876772, 1176038

[ref12] Chen P.-H. Chen R.-Y. Chou J.-Y. (2018). Screening and evaluation of yeast antagonists for biological control of *Botrytis cinerea* on strawberry fruits. Mycobiology 46, 33–46. doi: 10.1080/12298093.2018.1454013, 29998031 PMC6037076

[ref13] Cordero-Bueso G. Mangieri N. Maghradze D. Foschino R. Valdetara F. Cantoral J. M.. (2017). Wild grape-associated yeasts as promising biocontrol agents against *Vitis vinifera* fungal pathogens. Front. Microbiol. 8, 1–15. doi: 10.3389/fmicb.2017.02025, 29163377 PMC5675894

[ref14] De Ligne L. Vidal-Diez de Ulzurrun G. Baetens J. M. Van den Bulcke J. Van Acker J. De Baets B. (2019). Analysis of spatio-temporal fungal growth dynamics under different environmental conditions. IMA Fungus 10, 7–13. doi: 10.1186/s43008-019-0009-3, 32647616 PMC7325663

[ref15] Di Canito A. Mateo-vargas M. A. Mazzieri M. Cantoral J. Foschino R. Cordero-Bueso G.. (2021). The role of yeasts as biocontrol agents for pathogenic fungi on postharvest grapes: a review. Foods 10, 1–15. doi: 10.3390/foods10071650, 34359520 PMC8306029

[ref16] Di Francesco A. Ugolini L. Lazzeri L. Mari M. (2015). Production of volatile organic compounds by *Aureobasidium pullulans* as a potential mechanism of action against postharvest fruit pathogens. Biol. Control 81, 8–14. doi: 10.1016/j.biocontrol.2014.10.004

[ref21] FAO-OIV (2016). Food and Agriculture Organization, and Organisation Internationale de la Vine et du Vin focus 2016: table and dried grapes-non-alcoholic products of the vitivinicultural sector intended for human consumption, 1–64.

[ref17] Farbo M. G. Urgeghe P. P. Fiori S. Marcello A. Oggiano S. Balmas V.. (2018). Effect of yeast volatile organic compounds on ochratoxin A-producing *Aspergillus carbonarius* and *Aspergillus ochraceus*. Int. J. Food Microbiol. 284, 1–10. doi: 10.1016/j.ijfoodmicro.2018.06.023, 29990634

[ref18] Fernandez-San Millan A. Larraya L. Farran I. Ancin M. Veramendi J. (2021). Successful biocontrol of major postharvest and soil-borne plant *Pathogenic fungi* by *Antagonistic yeasts*. Biol. Control 160:104683. doi: 10.1016/j.biocontrol.2021.104683

[ref19] Ferraz P. Cássio F. Lucas C. (2019). Potential of yeasts as biocontrol agents of the Phytopathogen causing cacao witches’ broom disease: is microbial warfare a solution? Front. Microbiol. 10, 1–13. doi: 10.3389/fmicb.2019.01766, 31417539 PMC6685038

[ref20] Fleet G. H. Heard G. M. (1993). “Yeast growth during fermentation” in Wine Microbiology and Biotechnology. ed. G. H. Fleet (Harwood Academic: Switzerland), 27–54.

[ref22] Freimoser F. M. Rueda-Mejia M. P. Tilocca B. Migheli Q. (2019). Biocontrol yeasts: mechanisms and applications. World J. Microbiol. Biotechnol. 35, 154–119. doi: 10.1007/s11274-019-2728-4, 31576429 PMC6773674

[ref23] Hua S. S. T. Beck J. J. Sarreal S. B. L. Gee W. (2014). The major volatile compound 2-phenylethanol from the biocontrol yeast, *Pichia anomala*, inhibits growth and expression of aflatoxin biosynthetic genes of *Aspergillus flavus*. Mycotoxin Res. 30, 71–78. doi: 10.1007/s12550-014-0189-z, 24504634

[ref24] Huang R. Che H. J. Zhang J. Yang L. Jiang D. H. Li G. Q. (2012). Evaluation of *Sporidiobolus pararoseus* strain YCXT3 as biocontrol agent of *Botrytis cinerea* on post-harvest strawberry fruits. Biol. Control 62, 53–63. doi: 10.1016/J.BIOCONTROL.2012.02.010

[ref25] Huang R. Li G. Q. Zhang J. Yang L. Che H. J. Jiang D. H.. (2011). Control of postharvest botrytis fruit rot of strawberry by volatile organic compounds of *Candida intermedia*. Phytopathology 101, 859–869. doi: 10.1094/PHYTO-09-10-0255, 21323467

[ref26] Kassemeyer H.-H. (2017). “Fungi of grapes” in Biology of Microorganisms on Grapes, in Must and in Wine. eds. H. Konig, G. Unden and J. Frohlich (Heidelberg: Springer), 103–132.

[ref27] Köhl J. Kolnaar R. Ravensberg W. J. (2019). Mode of action of microbial biological control agents against plant diseases: relevance beyond efficacy. Front. Plant Sci. 10, 1–19. doi: 10.3389/fpls.2019.00845, 31379891 PMC6658832

[ref28] Leeder A. C. Palma-guerrero J. Glass N. L. (2011). The social network: deciphering fungal language. Nat. Rev. Microbiol. 9, 440–451. doi: 10.1038/nrmicro2580, 21572459

[ref29] Lemos Junior W. J. F. Bovo B. Nadai C. Crosato G. Carlot M. Favaron F.. (2016). Biocontrol ability and action mechanism of *Starmerella bacillaris* (synonym *Candida zemplinina*) isolated from wine musts against gray mold disease agent *Botrytis cinerea* on grape and their effects on alcoholic fermentation. Front. Microbiol. 7, 1–12. doi: 10.3389/fmicb.2016.01499, 27574517 PMC4983571

[ref30] Liu S. Laaksonen O. Li P. Gu Q. Yang B. (2022). Use of non-saccharomyces yeasts in berry wine production: inspiration from their applications in winemaking. J. Agric. Food Chem. 70, 736–750. doi: 10.1021/acs.jafc.1c07302, 35019274

[ref31] Maluleke E. Jolly N. P. Patterton H. G. Setati M. E. (2022). Antifungal activity of non-conventional yeasts against *Botrytis cinerea* and non-botrytis grape bunch rot fungi. Front. Microbiol. 13, 1–13. doi: 10.3389/fmicb.2022.986229, 36081805 PMC9445577

[ref32] Masoud W. Poll L. Jakobsen M. (2005). Influence of volatile compounds produced by yeasts predominant during processing of *Coffea arabica* in East Africa on growth and ochratoxin a (OTA) production by *Aspergillus ochraceus*. Yeast 22, 1133–1142. doi: 10.1002/yea.1304, 16240461

[ref33] Nally M. C. Pesce V. M. Maturano Y. P. Muñoz C. J. Combina M. Toro M. E.. (2012). Biocontrol of *Botrytis cinerea* in table grapes by non-pathogenic indigenous *Saccharomyces cerevisiae* yeasts isolated from viticultural environments in Argentina. Postharvest Biol. Technol. 64, 40–48. doi: 10.1016/j.postharvbio.2011.09.009

[ref34] Nally M. C. Pesce V. M. Maturano Y. P. Toro M. E. Combina M. Castellanos de Figueroa L. I.. (2013). Biocontrol of fungi isolated from sour rot infected table grapes by *Saccharomyces* and other yeast species. Postharvest Biol. Technol. 86, 456–462. doi: 10.1016/j.postharvbio.2013.07.022

[ref35] Nisiotou A. A. Nychas G. J. E. (2007). Yeast populations residing on healthy or botrytis-infected grapes from a vineyard in Attica, Greece. Appl. Environ. Microbiol. 73, 2765–2768. doi: 10.1128/AEM.01864-06, 17293525 PMC1855602

[ref36] OIV (2022). Organisation Internationale de la Vine et du Vin: State of the world vine and wine Sector 2021. 1–19.

[ref37] Oro L. Feliziani E. Ciani M. Romanazzi G. Comitini F. (2018). Volatile organic compounds from *Wickerhamomyces anomalus*, *Metschnikowia pulcherrima* and *Saccharomyces cerevisiae* inhibit growth of decay causing fungi and control postharvest diseases of strawberries. Int. J. Food Microbiol. 265, 18–22. doi: 10.1016/j.ijfoodmicro.2017.10.027, 29107842

[ref38] Palmieri D. Ianiri G. Del Grosso C. Barone G. De Curtis F. Castoria R.. (2022). Advances and perspectives in the use of biocontrol agents against fungal plant diseases. Horticulturae 8:577. doi: 10.3390/horticulturae8070577

[ref39] Pantelides I. S. Christou O. Tsolakidou M. D. Tsaltas D. Ioannou N. (2015). Isolation, identification and in vitro screening of grapevine yeasts for the control of black aspergilli on grapes. Biol. Control 88, 46–53. doi: 10.1016/j.biocontrol.2015.04.021

[ref40] Parafati L. Vitale A. Restuccia C. Cirvilleri G. (2015). Biocontrol ability and action mechanism of food-isolated yeast strains against *Botrytis cinerea* causing post-harvest bunch rot of table grape. Food Microbiol. 47, 85–92. doi: 10.1016/j.fm.2014.11.013, 25583341

[ref41] Pereyra M. M. Díaz M. A. Soliz-Santander F. F. Poehlein A. Meinhardt F. Daniel R.. (2021). Screening methods for isolation of biocontrol *Epiphytic* yeasts against *Penicillium digitatum* in lemons. J. Fungi 7:166. doi: 10.3390/jof7030166, 33669096 PMC7996618

[ref42] Pertot I. Caffi T. Rossi V. Mugnai L. Hoffmann C. Grando M. S.. (2017a). A critical review of plant protection tools for reducing pesticide use on grapevine and new perspectives for the implementation of IPM in viticulture. Crop Prot. 97, 70–84. doi: 10.1016/j.cropro.2016.11.025

[ref43] Pertot I. Giovannini O. Benanchi M. Caffi T. Rossi V. Mugnai L. (2017b). Combining biocontrol agents with different mechanisms of action in a strategy to control *Botrytis cinerea* on grapevine. Crop Prot. 97, 85–93. doi: 10.1016/j.cropro.2017.01.010

[ref44] Petruzzi L. Capozzi V. Berbegal C. Corbo M. R. Bevilacqua A. Spano G.. (2017). Microbial resources and enological significance: opportunities and benefits. Front. Microbiol. 8, 1–13. doi: 10.3389/fmicb.2017.00995, 28642742 PMC5462979

[ref45] Pielou E. C. (1966). The measurement of diversity in different types of biological collections. J. Theor. Biol. 13, 131–144. doi: 10.1016/0022-5193(66)90013-0

[ref46] Pinto C. Pinho D. Cardoso R. Custódio V. Fernandes J. Sousa S.. (2015). Wine fermentation microbiome: a landscape from different Portuguese wine appellations. Front. Microbiol. 6, 1–13. doi: 10.3389/fmicb.2015.00905, 26388852 PMC4555975

[ref47] Poitevin C. G. Veronesi R. S. Pimentel I. C. Auer C. G. (2020). Foliar application of endophytic *Wickerhamomyces anomalus* against grey mould in *Eucalyptus dunnii*. Biocontrol Sci. Tech. 30, 93–102. doi: 10.1080/09583157.2019.1687645

[ref49] Pretscher J. Fischkal T. Branscheidt S. Jäger L. Kahl S. Schlander M.. (2018). Yeasts from different habitats and their potential as biocontrol agents. Fermentation 4:31. doi: 10.3390/fermentation4020031

[ref50] Raspor P. Miklič-Milek D. Avbelj M. Čadež N. (2010). Biocontrol of grey mould disease on grape caused by *Botrytis cinerea* with autochthonous wine yeasts. Food Technol. Biotechnol. 48, 336–343.

[ref51] Reyes-bravo P. Acuña-fontecilla A. Rosales I. M. I. M. Godoy L. (2019). Evaluation of native wine yeast as biocontrol agents against fungal pathogens related to postharvest diseases. Agric. Sci. Agron. doi: 10.20944/preprints201909.0113.v1

[ref52] Romo-Sánchez S. Alves-Baffi M. Arévalo-Villena M. Úbeda-Iranzo J. Briones-Pérez A. (2010). Yeast biodiversity from oleic ecosystems: study of their biotechnological properties. Food Microbiol. 27, 487–492. doi: 10.1016/j.fm.2009.12.009, 20417397

[ref53] Scheff J. D. Almon R. R. Dubois D. C. Jusko W. J. Androulakis I. P. (2011). Assessment of pharmacologic area under the curve when baselines are variable. Pharm. Res. 28, 1081–1089. doi: 10.1007/s11095-010-0363-8, 21234658 PMC3152796

[ref54] Seixas I. Barbosa C. Mendes-faia A. Mendes-ferreira A. Mira N. P. (2019). Genome sequence of the non-conventional wine yeast *Hanseniaspora guilliermondii* UTAD222 unveils relevant traits of this species and of the *Hanseniaspora genus* in the context of wine fermentation. DNA Res. 26, 67–83. doi: 10.1093/dnares/dsy039, 30462193 PMC6379042

[ref55] Serra R. Braga A. Venâncio A. (2005). Mycotoxin-producing and other fungi isolated from grapes for wine production, with particular emphasis on ochratoxin a. Res. Microbiol. 156, 515–521. doi: 10.1016/j.resmic.2004.12.005, 15862450

[ref56] Sipiczki M. (2006). *Metschnikowia* strains isolated from botrytized grapes antagonize fungal and bacterial growth by iron depletion. Appl. Environ. Microbiol. 72, 6716–6724. doi: 10.1128/AEM.01275-06, 17021223 PMC1610289

[ref57] Sipiczki M. (2022). Taxonomic revision of the pulcherrima clade of *Metschnikowia* (fungi): merger of species. Taxon 2, 107–123. doi: 10.3390/taxonomy2010009

[ref58] Sirén K. Mak S. S. T. Melkonian C. Carøe C. Swiegers J. H. Molenaar D.. (2019). Taxonomic and functional characterization of the microbial community during spontaneous in vitro fermentation of Riesling must. Front. Microbiol. 10, 1–17. doi: 10.3389/fmicb.2019.00697, 31024486 PMC6465770

[ref59] Solairaj D. Yang Q. Guillaume Legrand N. N. Routledge M. N. Zhang H. (2021). Molecular explication of grape berry-fungal infections and their potential application in recent postharvest infection control strategies. Trends Food Sci. Technol. 116, 903–917. doi: 10.1016/j.tifs.2021.08.037

[ref60] Song G. C. Ryu C. (2013). Two volatile organic compounds trigger plant self-defense against a bacterial pathogen and a sucking insect in cucumber under open field conditions. Int. J. Mol. Sci. 14, 9803–9819. doi: 10.3390/ijms14059803, 23698768 PMC3676814

[ref61] Spadaro D. Droby S. (2016). Development of biocontrol products for postharvest diseases of fruit: the importance of elucidating the mechanisms of action of yeast antagonists. Trends Food Sci. Technol. 47, 39–49. doi: 10.1016/j.tifs.2015.11.003

[ref62] Stenberg J. A. Sundh I. Becher P. G. Björkman C. Dubey M. Egan P. A.. (2021). When is it biological control? A framework of definitions, mechanisms, and classifications. J. Pest. Sci. 94, 665–676. doi: 10.1007/s10340-021-01354-7

[ref63] Strauss M. L. A. Jolly N. P. Lambrechts M. G. Van Rensburg P. (2001). Screening for the production of extracellular hydrolytic enzymes by non-saccharomyces wine yeasts. J. Appl. Microbiol. 91, 182–190. doi: 10.1046/j.1365-2672.2001.01379.x, 11442729

[ref64] Suzzi G. Romano P. Ponti I. Montuschi C. (1995). Natural wine yeasts as biocontrol agents. J. Appl. Bacteriol. 78, 304–308. doi: 10.1111/j.1365-2672.1995.tb05030.x

[ref65] Swadling I. R. Jeffries P. (1996). Isolation of microbial antagonists for biocontrol of grey mould disease of strawberries. Biocontrol Sci. Tech. 6, 125–136. doi: 10.1080/09583159650039584

[ref66] Tilocca B. Cao A. Migheli Q. (2020). Scent of a killer: microbial Volatilome and its role in the biological control of plant pathogens. Front. Microbiol. 11:41. doi: 10.3389/fmicb.2020.00041, 32117096 PMC7018762

[ref67] Vicente J. Navascués E. Calderón F. Santos A. Marquina D. Benito S. (2021). An integrative view of the role of Lachancea thermotolerans in wine technology. Foods 10:2878. doi: 10.3390/foods10112878 34829158 PMC8625220

[ref68] Ward M. G. (2016). The regulatory landscape for biological control agents. Bull. OEPP/EPPO 46, 249–253. doi: 10.1111/epp.12307

[ref69] Wilson C. L. Wisniewski M. (1989). Biological control of post harvest diseases offruit and vegetables: an emerging technology. Annu. Rev. Phytopathol. 27, 425–441. doi: 10.1146/annurev.py.27.090189.002233

[ref70] Wobbrock J. O. Findlater L. Gergle D. Higgins J. J. (2011). “The aligned rank transform for nonparametric factorial analyses using only ANOVA procedures” in Proceedings of the SIGCHI Conference on Human Factors in Computing Systems. New York: Association for Computer Machinery, 143–146.

[ref71] Zar J. H. (1996). Biostatistical Analysis. New Jersey, USA: Prentice Hall Inc.

[ref72] Zhang H. Godana E. A. Sui Y. Yang Q. Zhang X. Zhao L. (2020). Biological control as an alternative to synthetic fungicides for the management of grey and blue mould diseases of table grapes: a review. Crit. Rev. Microbiol. 46, 450–462. doi: 10.1080/1040841X.2020.1794793, 32730726

