# Correction: Biocontrol potential of wine yeasts against four grape phytopathogenic fungi disclosed by time-course monitoring of inhibitory activities

**DOI:** 10.3389/fmicb.2026.1817348

**Published:** 2026-05-01

**Authors:** Marcos Esteves, Patrícia Lage, João Sousa, Filipe Centeno, Maria de Fátima Teixeira, Rogério Tenreiro, Ana Mendes-Ferreira

**Affiliations:** 1 WM&B—Laboratory of Wine Microbiology and Biotechnology, Department of Biology and Environment, University of Trás-os-Montes and Alto Douro, Vila Real, Portugal; 2 BioISI—Biosystems & Integrative Sciences Institute, Faculty of Sciences, University of Lisbon, Lisbon, Portugal; 3 PROENOL—Indústria Biotecnológica, Lda, Canelas, Portugal

**Keywords:** biocontrol, wine yeasts, phytopathogenic fungi, antagonism, *Aspergillus niger*, *Botrytis cinerea*, *Penicillium* sp., *Rhizopus* sp.

In the published article, there was an error in the taxonomic identification of the isolate *Mucor* sp. MU3. Subsequent molecular identification revealed that MU3 isolate belongs to the *Rhizopus* genus.

Corrections have been made throughout the text, replacing “*Mucor* sp. MU3” by “*Rhizopus* sp. MU3”.

A correction has been made to **Discussion**, Paragraph 5, with the removal of the following sentences:

“Also, to our knowledge, our study is the first showing the potential of wine yeasts to control *Mucor* sp. development. We were only able to find one study on the biocontrol activity of yeasts against *Mucor* rot of pears, by a pear-isolated strain of *Cryptococcus infirmo-miniatus* (Chand-Goyal and Spotts, 1996).”

The corrected identification (*Rhizopus* sp. MU3) invalidates the claim of novelty when applied to the inhibition of *Rhizopus* species (Nally et al., 2015; Delgado et al., 2021).

The original article has been updated.

