# Supplementary material for: Biocontrol potential of wine yeasts against four grape phytopathogenic fungi disclosed by time-course monitoring of inhibitory activities
Source: Front Microbiol. 2023 Mar 7;14:1146065. doi: 10.3389/fmicb.2023.1146065 (PMC10028181; doi:10.3389/fmicb.2023.1146065)
Supplement: Supplementary file 2 [file Image_1.pdf]

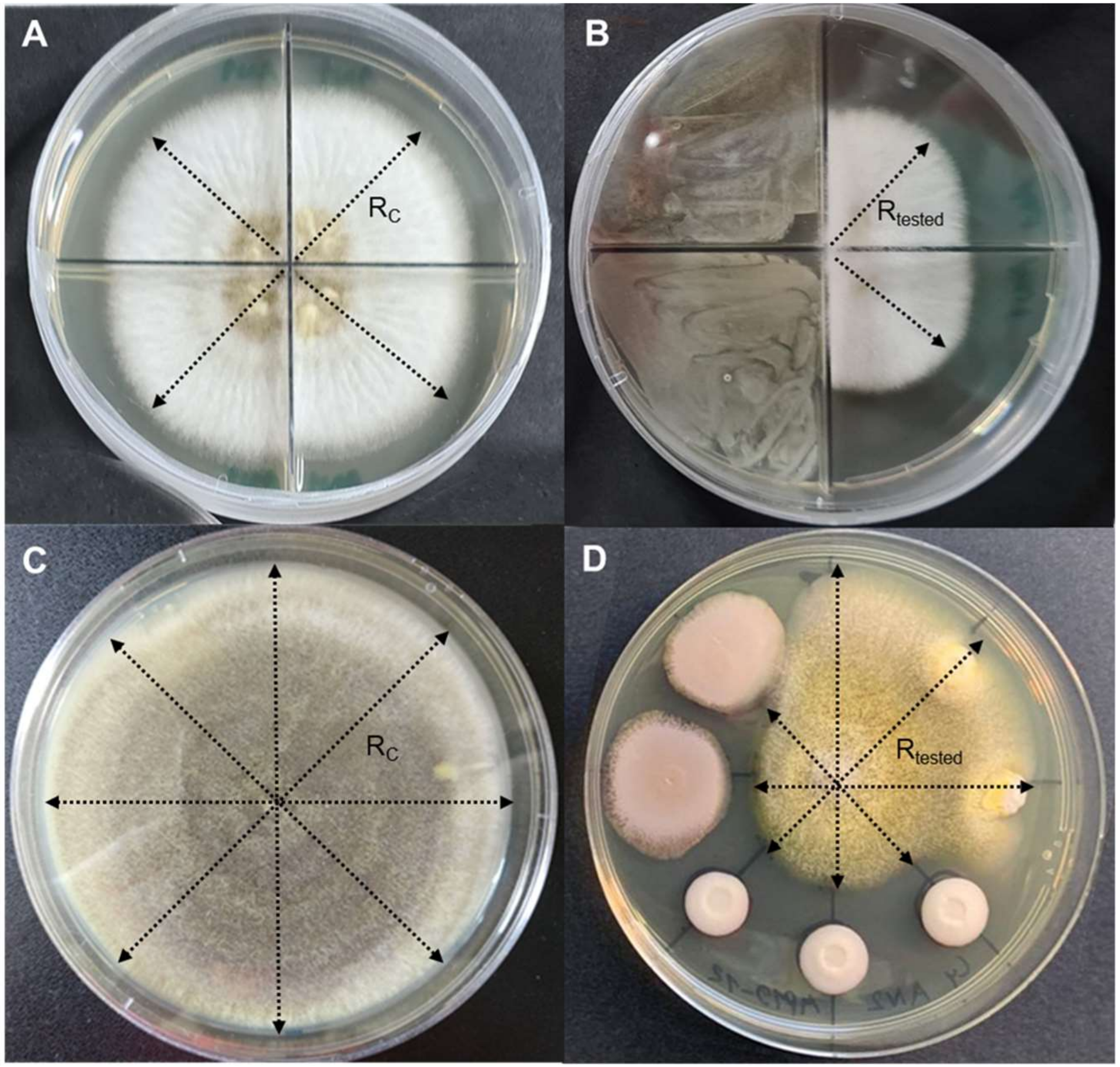

Figure S1: **Schematic overview and examples of the in *vitro* antagonistic activity assays**

To screen for yeasts producing antimicrobial volatiles, petri dishes were used with separated compartments (A and B). The yeast strain was seeded in two compartments and the target was inoculated at the corner of the other two compartments of the Petri dish (B). In mycelial growth inhibition assay (C and D), both the yeast strain and the target were spotted on the solid agar medium. The target was spotted at a 3 cm distance from the yeast strain. As a control (A, C) Petri dishes without the inoculation of yeasts were used. Radial mycelia growth was measured where $R_{control}$ and R_{tested} represents the fungal mycelium radius in the absence of yeast (A and C) and with yeast (B and D), respectively.
