# Supplementary material for: Biocontrol potential of wine yeasts against four grape phytopathogenic fungi disclosed by time-course monitoring of inhibitory activities
Source: Front Microbiol. 2023 Mar 7;14:1146065. doi: 10.3389/fmicb.2023.1146065 (PMC10028181; doi:10.3389/fmicb.2023.1146065)
Supplement: Supplementary file 3 [file Image_2.pdf]

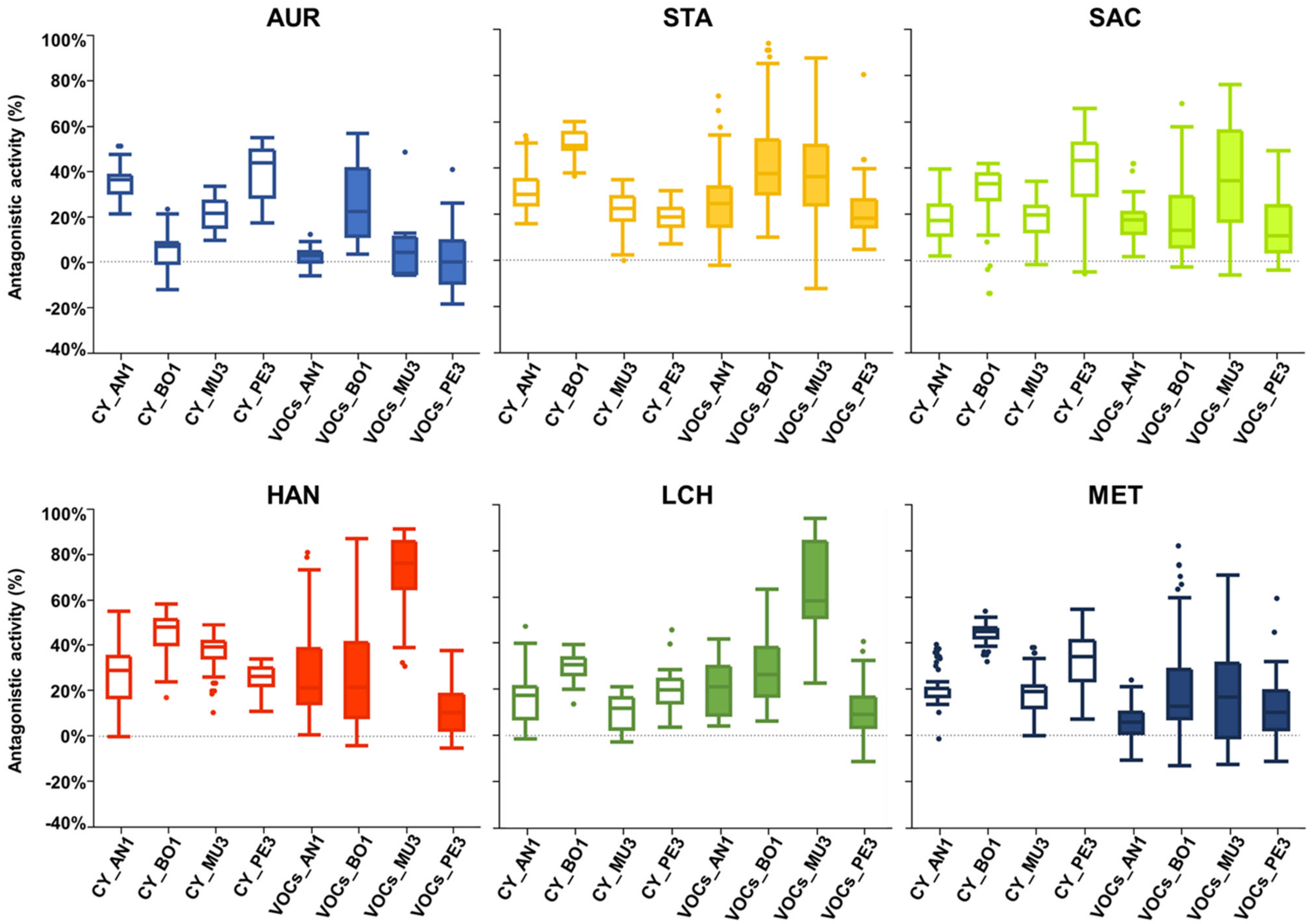

Fig.S2 – Box-plots of yeast antagonistic activity against the four fungal targets. For the most representative yeast genera, *Aureobasidium* (AUR), *Starmerella* (STA), *Lachancea* (LCH), *Hanseniaspora* (HAN), *Saccharomyces* (SAC) and *Metschnikowia* (MET**),** it is represented the distribution of fungal targets response mediated by diffusible (CY) and volatile compounds (VOCs), determined by IRG (%). AN1 - *Aspergillus niger*; BO1 - *Botrytis cinerea*; MU3 - *Mucor* sp.; PE3 - *Penicillium* sp.

.
