# Supplementary material for: Biocontrol potential of wine yeasts against four grape phytopathogenic fungi disclosed by time-course monitoring of inhibitory activities
Source: Front Microbiol. 2023 Mar 7;14:1146065. doi: 10.3389/fmicb.2023.1146065 (PMC10028181; doi:10.3389/fmicb.2023.1146065)
Supplement: Supplementary file 4 [file Image_3.pdf]

Figure S3: **Hierarchical clustering of fungal growth profiles for each fungal target in each assay (CY and VOCs).** Dendrograms were obtained by hierarchical analysis of growth profile patterns of *Aspergillus niger* AN1, *Botrytis cinerea* BO1, *Mucor* sp. MU3 and *Penicillium* sp. PE3 in CY and VOCs assays. using Pearson's correlation coefficient and the UPGMA clustering method. The average profiles defined at 80% of similarity (B) were subsequently visually inspected to define the representative growth patterns for each target which are presented in Figure 3 . The fungal growth profile in the absence of yeast (control) is represented in red.

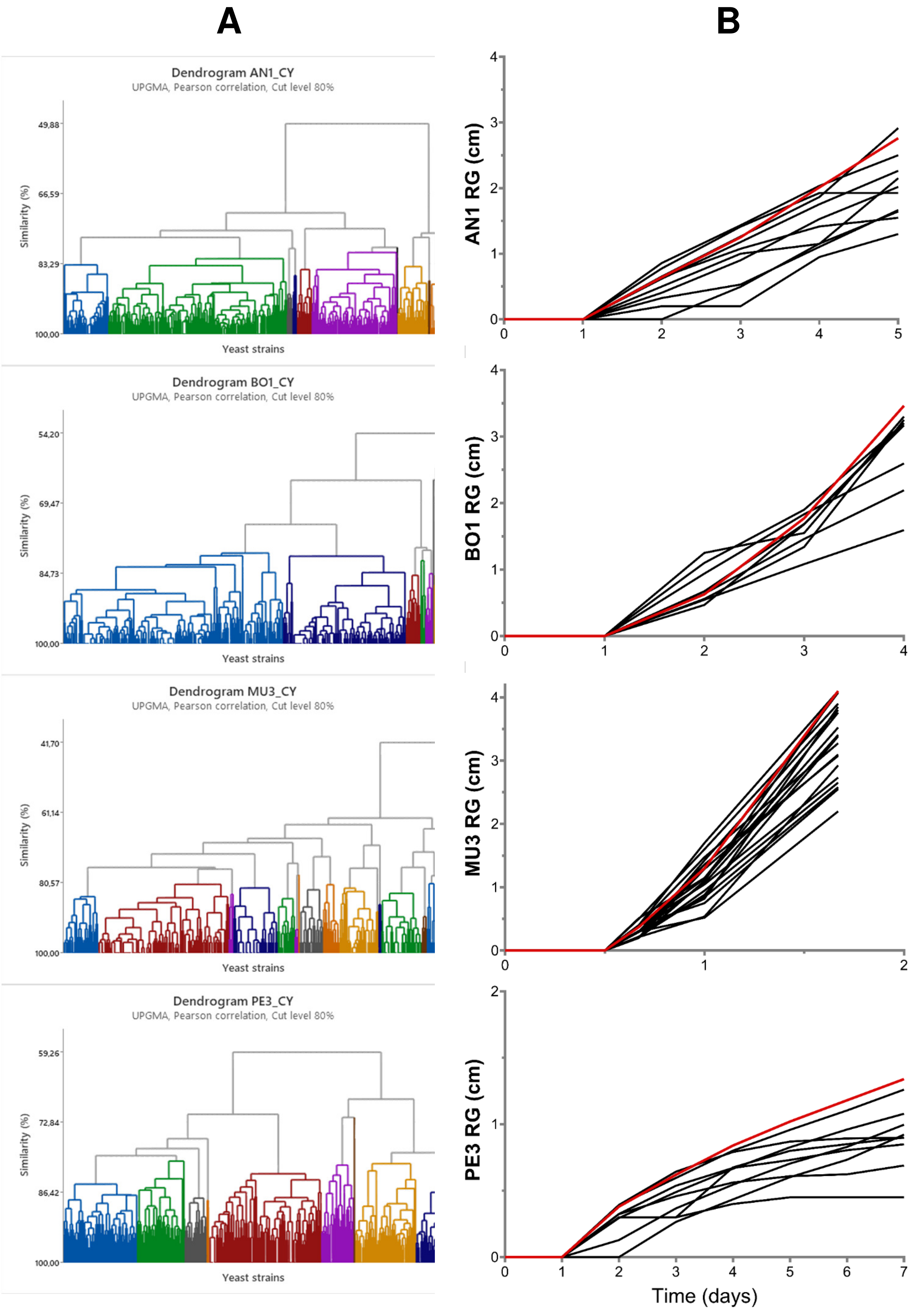

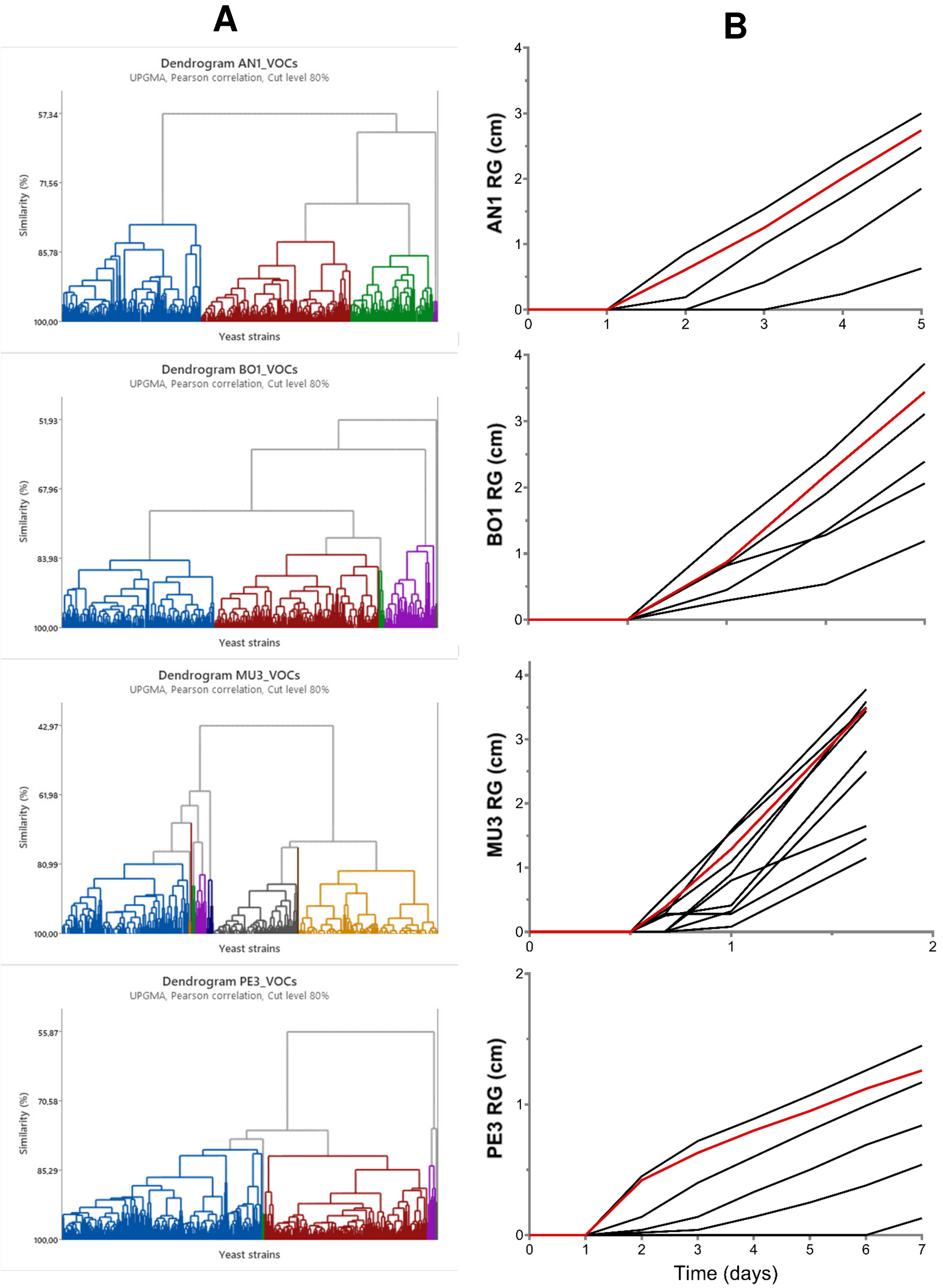
A
B
Dendrogram AN1_VOCs
UPGMA, Pearson correlation, Cut level 80%
Similarity (%)
57,34
71,56
85,78
100,00
Yeast strains
Dendrogram BO1_VOCs
UPGMA, Pearson correlation, Cut level 80%
Similarity (%)
51,93
67,96
83,98
100,00
Yeast strains
Dendrogram MU3_VOCs
UPGMA, Pearson correlation, Cut level 80%
Similarity (%)
42,97
61,98
80,99
100,00
Yeast strains
Dendrogram PE3_VOCs
UPGMA, Pearson correlation, Cut level 80%
Similarity (%)
55,87
70,58
85,29
100,00
Yeast strains
AN1 RG (cm)
BO1 RG (cm)
MU3 RG (cm)
PE3 RG (cm)
Time (days)
