# Supplementary material for: Biocontrol potential of wine yeasts against four grape phytopathogenic fungi disclosed by time-course monitoring of inhibitory activities
Source: Front Microbiol. 2023 Mar 7;14:1146065. doi: 10.3389/fmicb.2023.1146065 (PMC10028181; doi:10.3389/fmicb.2023.1146065)
Supplement: Supplementary file 5 [file Image_4.pdf]

**Figure S4. Genomic diversity of the most representative genera.** Dendrograms of *Aureobasidium* (AUR), *Starmerella* (STA), *Lachancea* (LCH), *Hanseniaspora* (HAN), *Saccharomyces* (SAC) and *Metschnikowia* (MET) isolates obtained by composite hierarchical analysis of polymerase chain reaction (PCR) $(GTG)_5$ and M13 patterns using Pearson's correlation coefficient and the UPGMA clustering method. The genomic groups of strains were defined at 85% similarity. The number of isolates assigned to each cluster is displayed.

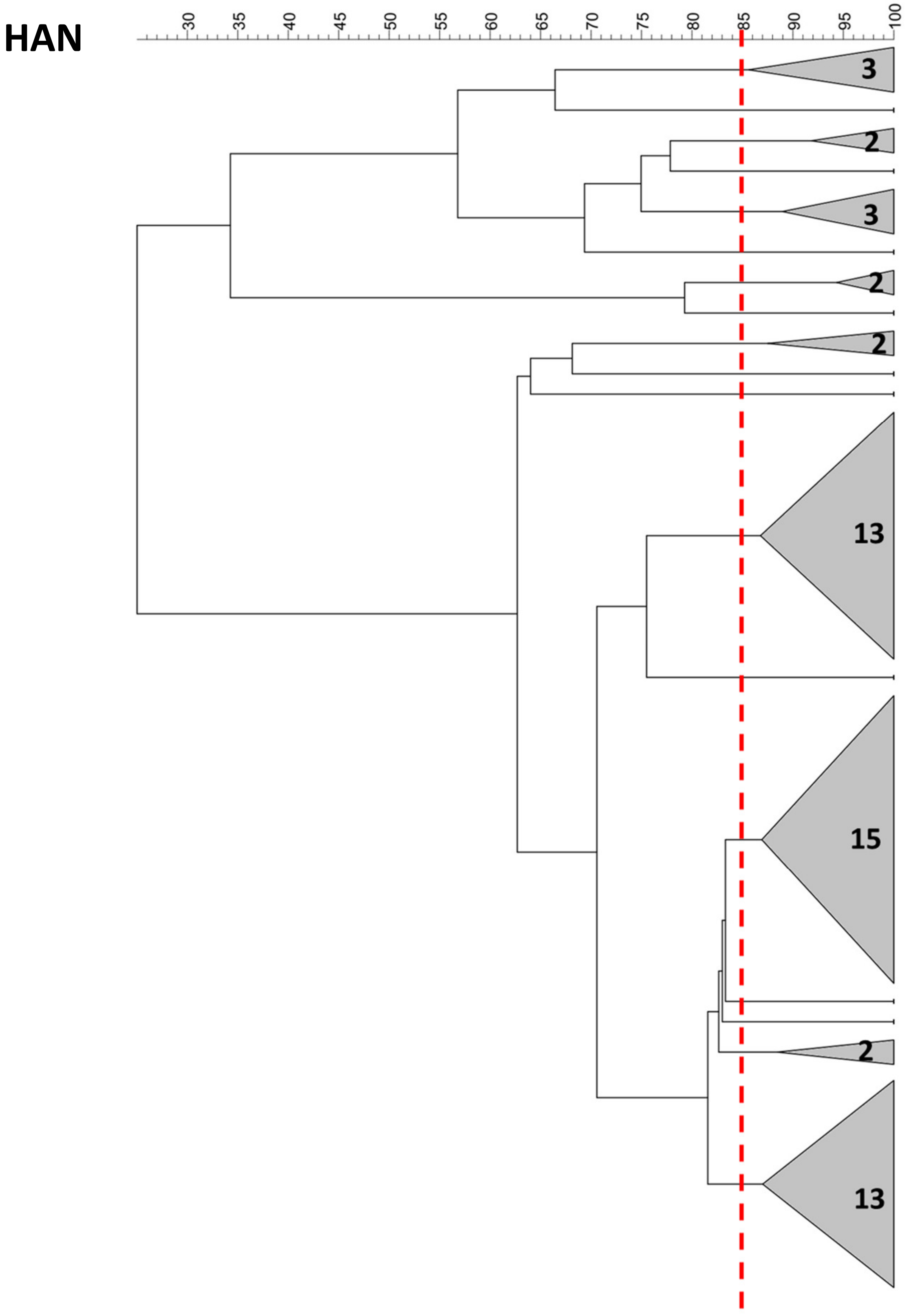

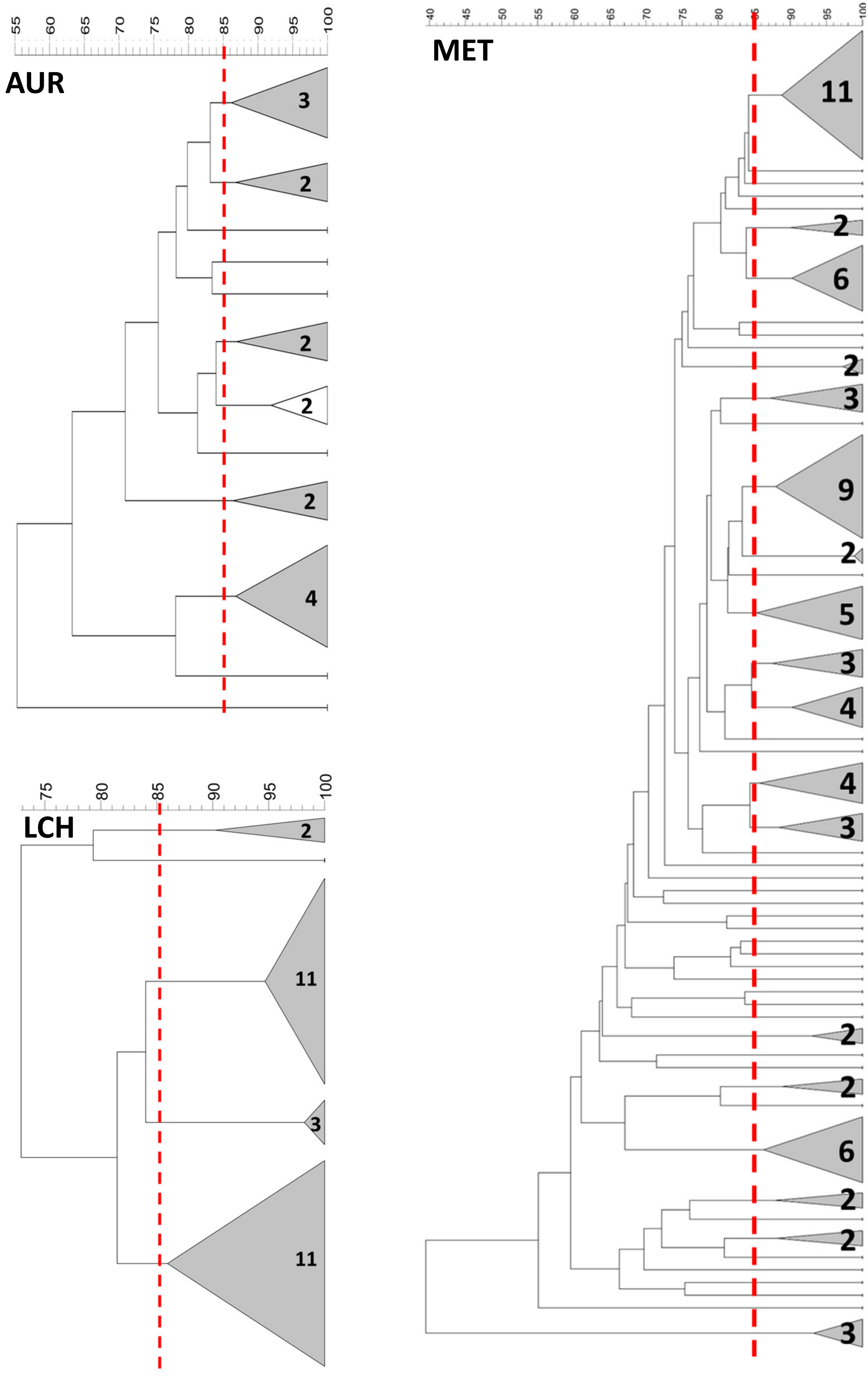
AUR
55 60 65 70 75 80 85 90 95 100
3
2
2
2
2
4
LCH
75 80 85 90 95 100
2
11
3
11
MET
40 45 50 55 60 65 70 75 80 85 90 95 100
11
2
6
2
3
9
2
5
3
4
4
3
2
2
6
2
2
3

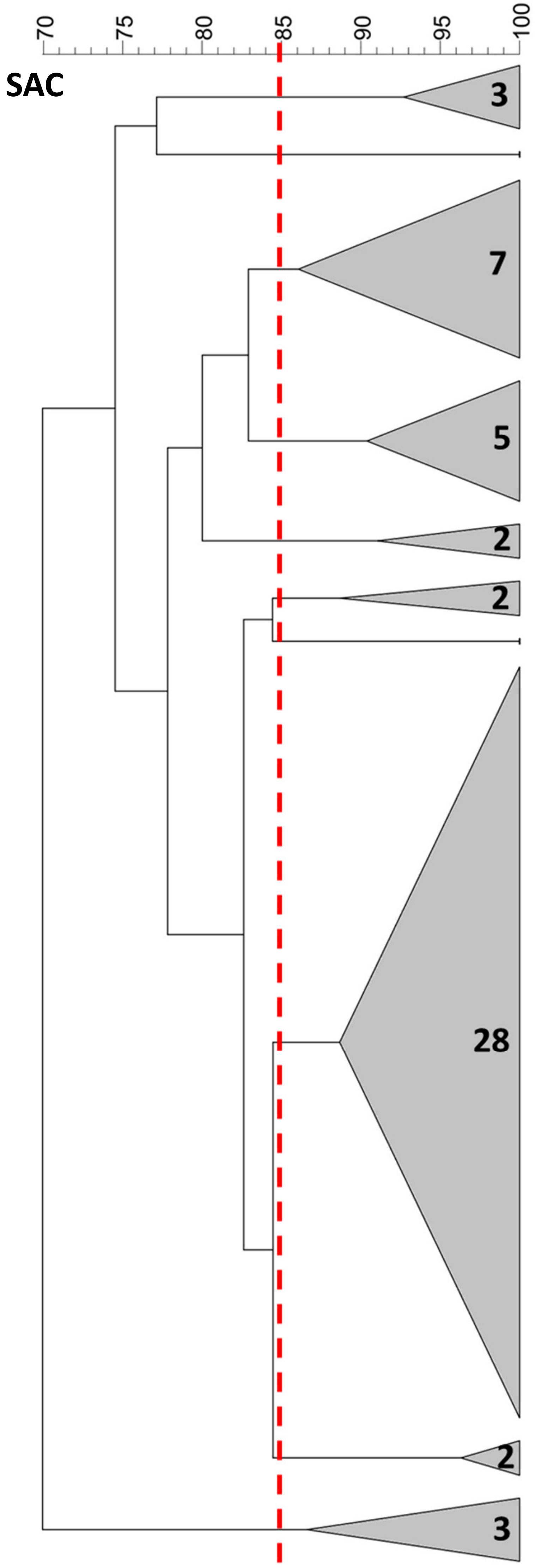

SAC
70
75
80
85
90
95
100
3
7
5
2
2
28
2
3

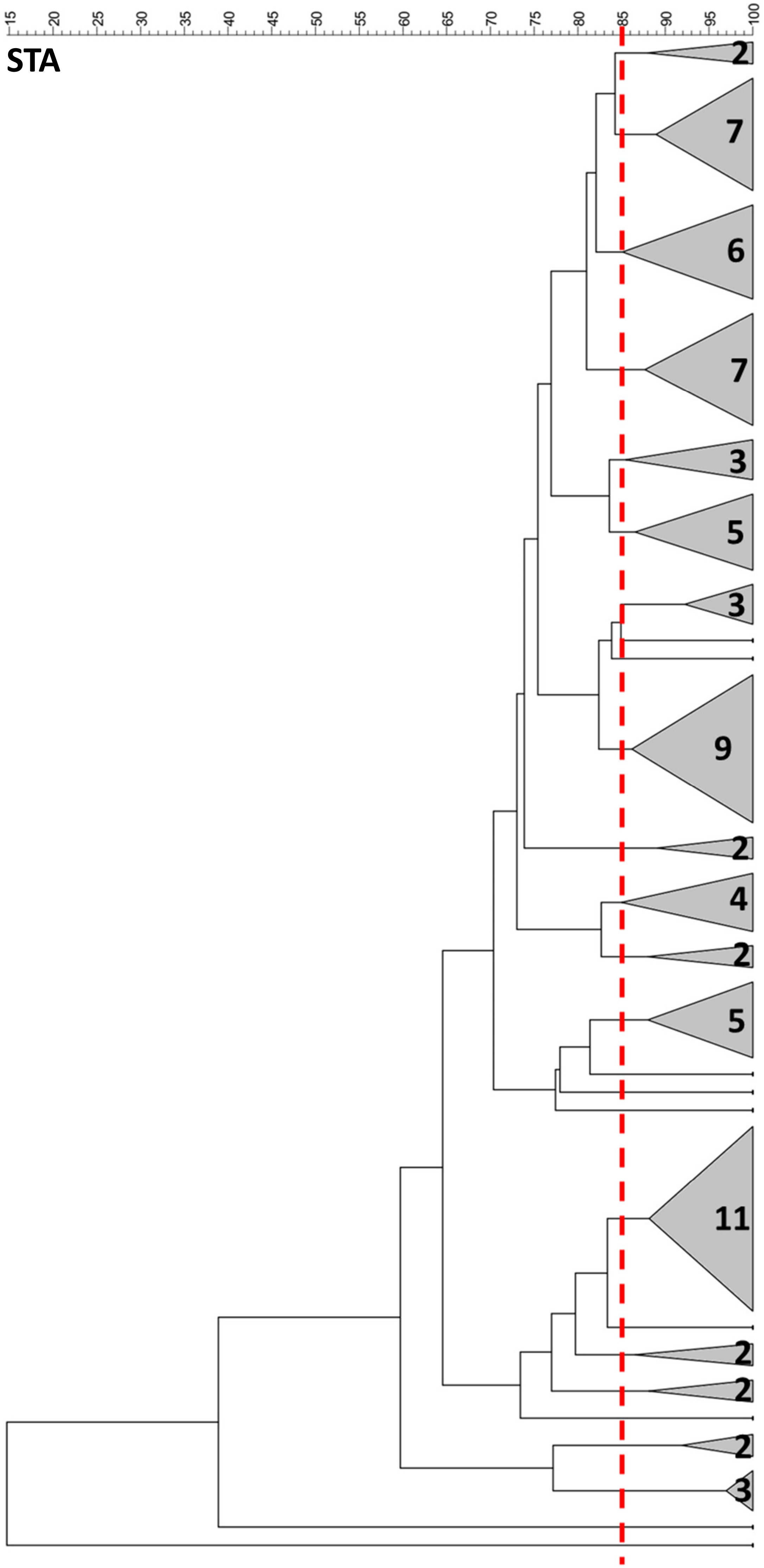
STA
15 20 25 30 35 40 45 50 55 60 65 70 75 80 85 90 95 100
2
7
6
7
3
5
3
9
2
4
2
5
11
2
2
2
3
