# Supplementary material for: Biocontrol potential of wine yeasts against four grape phytopathogenic fungi disclosed by time-course monitoring of inhibitory activities
Source: Front Microbiol. 2023 Mar 7;14:1146065. doi: 10.3389/fmicb.2023.1146065 (PMC10028181; doi:10.3389/fmicb.2023.1146065)
Supplement: Supplementary file 6 [file Image_5.pdf]

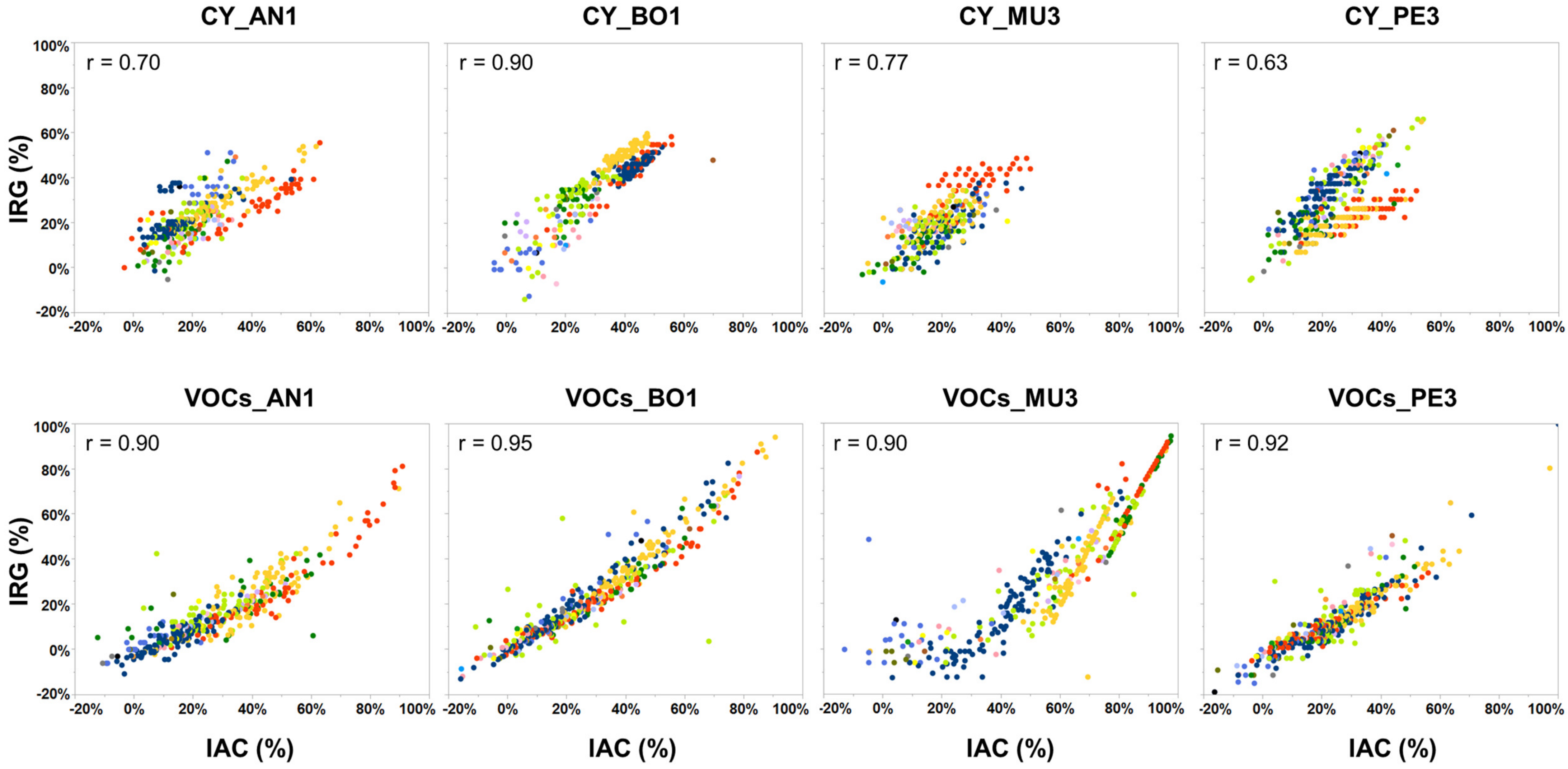

Figure S5. Comparison of yeast antagonistic activity against the four fungal targets derived from IRG and IAC parameters. The spatial representation of the 397 yeast strains built on their inhibitory activity against *Aspergillus niger* AN1, *Botrytis cinerea* BO1, *Mucor* sp. MU3 and *Penicillium* sp. PE3, mediated by diffusible (CY) and volatile compounds (VOCs) and determined by IAC or IRG parameters. The Pearson correlation coefficient (r) between IAC and IRG parameters is presented. The colors represent the different genera affiliation of the yeast strains following the color scheme used in Figure 1.
