# Supplementary material for: Biocontrol potential of wine yeasts against four grape phytopathogenic fungi disclosed by time-course monitoring of inhibitory activities
Source: Front Microbiol. 2023 Mar 7;14:1146065. doi: 10.3389/fmicb.2023.1146065 (PMC10028181; doi:10.3389/fmicb.2023.1146065)
Supplement: Supplementary file 7 [file Image_6.pdf]

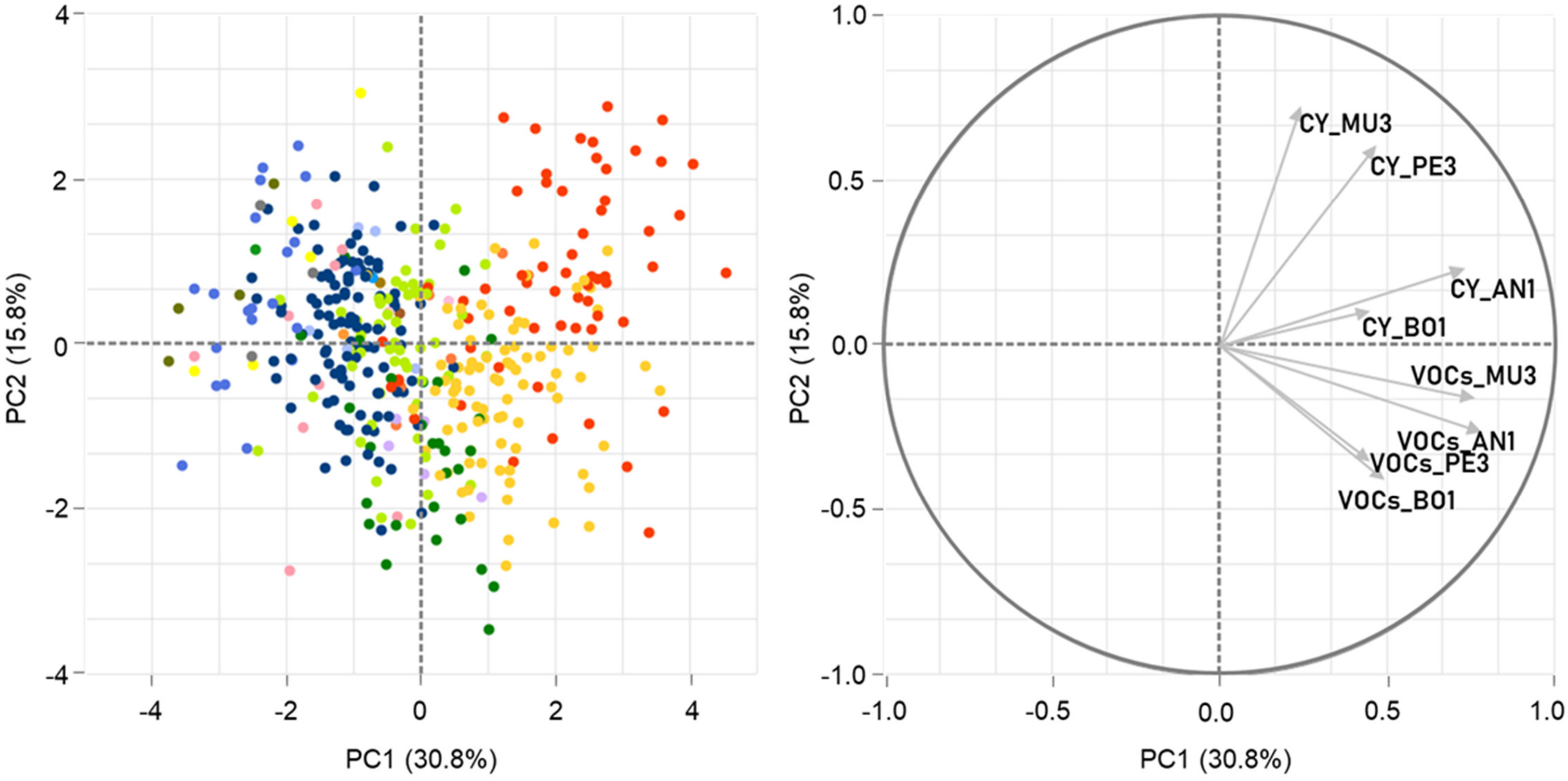

Figure S6. Principal component analysis (PCA) of yeast antagonistic activity against the four fungal targets. The spatial representation of the 397 yeast strains according to the two first principal components (PC1 and PC2) is built on their inhibitory activity against *Aspergillus niger* AN1, *Botrytis cinerea* BO1, *Mucor* sp. MU3 and *Penicillium* sp. PE3, mediated by diffusible (CY) and volatile compounds (VOCs) and determined by IAC (%). The data points, corresponding to the yeast strains tested, were colored by genera affiliation following the color scheme used in Figure1.
